# Research on the pore distribution characteristics and strength degradation of cement-based materials under sulfate attack

**DOI:** 10.1038/s41598-025-31233-5

**Published:** 2025-12-07

**Authors:** Yuhang Li, Enze Hao, Xiumei Zheng, Dali Zhang, Wenbang Zhu, Ruiming Liu, Xinjie Wang, Yali Cao, Chaochao Sun, Debo He, Yang Chao, Gang Li

**Affiliations:** 1 College of Civil Engineering, Kashi University, Kashi, 844006 China; 2 Xinjiang Key Laboratory of Engineering Materials and Structural Safety, kashi, 844006 China; 3 Liaoning Construction Science Research Institute Co, Shenyang, 110000 China

**Keywords:** Sulfate corrosion, Cement-based materials, Porosity, Quality change, Compressive strength, Engineering, Materials science

## Abstract

To investigate the influence of sulfate erosion on the performance of cement-based materials, this study designed cement paste, mortar and concrete specimens with a water-to-cement ratio of 0.35. The study examined the changes in performance over 30 days, 60 days, 90 days, 120 days, and 150 days of sulfate erosion, and the findings showed that: as the duration of sulfate erosion increased, the mass change and compressive strength of cement-based materials generally showed an increasing trend first and then decreasing trend. The mass change of cement paste, mortar and concrete specimens was 0.64%, 0.274%, and 0.16%, respectively, after 150 days of sulfate erosion. The compressive strength of the 150-day cement paste, mortar, and concrete decreased by 56.86%, 14.29%, and 19.34% respectively.The porosity of cement paste, mortar and concrete specimens showed that the porosity of cement paste was greater than that of concrete, which was greater than that of mortar specimens.When employing the Mercury Intrusion Porosimetry (MIP) test, the porosity of mortar specimens decreased initially and then increased with prolonged sulfate exposure. The porosity of mortar specimens reached 12.57% after 30 days of sulfate erosion and 18.27% after 150 days of sulfate erosion.When Nuclear Magnetic Resonance (NMR) testing was adopted, the variation law of porosity after sulfate attack was basically consistent with that of MIP testing, but there were differences in porosity. The porosity was 7.60% after 30 days of erosion and 14.42% after 150 days of erosion. A comprehensive comparison of the two testing methods revealed numerical differences, but it could provide a more comprehensive understanding of the influence mechanism of pore structure on the performance of cement-based materials, and offer important references for optimizing material ratios and improving material performance.

**Supplementary Information:**

The online version contains supplementary material available at 10.1038/s41598-025-31233-5.

## Introduction

Cement-based materials are the most extensively utilized construction materials globally, offering advantages such as ease of availability, cost-effectiveness, and excellent fire resistance. They find widespread application in residential buildings, bridges, roads, and various other structures^1^. Under standard conditions, cement-based materials exhibit commendable performance characteristics including compressive strength^2–4^, deformation capacity^5,6^, and durability^7–9^. However, due to their specific geographical locations or environmental conditions, structures situated in coastal regions^10^ and saline-alkali areas are subjected to prolonged exposure to factors such as saline-alkali solutions and steam. Among these influences, sulfate erosion is one of the more prevalent degradation mechanisms^11^. In such scenarios, sulfate erosion can compromise several mechanical properties of cement-based materials; consequently, for relevant construction projects and marine engineering applications affected by sulfate attack on cement-based materials, the overall structural durability may be diminished. This reduction poses significant risks to both life safety and property.

The sulfate attack on cement-based materials can be primarily categorized into physical and chemical reactions^12–14^. Physical reaction pertains to the crystallization of sulfates due to water evaporation and other factors during the sulfate erosion process^15^. Chemical reaction involves the interaction between sulfates and chemical constituents in cement-based materials, resulting in the formation of gypsum, ettringite, thaumasite, among others^16,17^. The degradation of concrete by sulfates is predominantly manifested through damage to hydrated calcium silicate gel, leading to a disruption of the original internal structural equilibrium of concrete^18^. Sulfate attacks typically present themselves in concrete as expansion and cracking phenomena^19,20^. When cracks develop, the accompanying sulfate solution is more prone to infiltrating the internal pores of concrete^21^, adversely affecting key properties such as strength and durability post-sulfate attack. Under these circumstances, sulfate exposure diminishes the strength of cement-based materials^22,23^. Consequently, for construction projects and marine engineering endeavors situated in saline environments, sulfate-induced erosion poses a significant threat to overall structural durability by compromising cement-based material integrity—thereby endangering life safety and property.

Currently, substantial advancements have been made regarding understanding how sulfates affect concrete erosion. Research conducted by Chindaprasirt et al.^24^ and Gao et al.^25^ examined damage characteristics under dry-wet cycling conditions—including ion diffusion patterns, volumetric expansion behaviors, weight loss metrics, alterations in mechanical properties—and confirmed that wet-dry cycling significantly influences concrete’s damage progression compared with isolated sulfate environments. Meng et al.^26^ investigated high-performance synthetic fiber-reinforced concrete subjected to sulfate erosion; their findings indicated superior crack resistance and freeze-thaw resilience relative to conventional concrete types. Tan et al.^27^ analyzed load impacts on concrete columns through compression testing while discussing how sulfates influence these structures’ performance attributes. Utilizing an extended numerical approach based on Ficks second law alongside second-order reaction dynamics principles, Wang et al.^28^ concentrated on developing numerical analytical simulations along with ion diffusion models under sulfuric action aimed at predicting erosive processes within sodium sulfate solutions impacting concretes.In addition, some scholars have explored corresponding studies concerning crack morphology within concretes exposed to Sulfate attacks, and multi-faceted environmental changes induced by Sulfate under complex conditions^29–31^.

However, the current analytical methods for studying the dynamic impact of sulfate on concrete mostly adopt Scanning Electron Microscopy (SEM) or X-ray Diffraction (XRD)^14,32^ for analysis and interpretation from a microscopic perspective. Although these methods can reveal the microstructure changes and phase composition of cement-based materials after sulfate attack, they are insufficient in characterizing the key indicator of pore distribution features, especially the dynamic changes in pore size, shape, and connectivity. As two non-destructive pore structure characterization techniques, MIP and NMR can provide detailed information on the pore distribution of cement-based materials at different scales^33–35^. MIP acquires the pore size distribution by measuring the volume of mercury that invades the material pores at different pressures, mainly revealing the ‘throat’ or ‘entrance’ size of the pore network. It is unable to detect closed pores, dead-end pores, etc., which leads to limitations in understanding the total pore volume and pore connectivity evolution. To fill this gap, this study for the first time combines MIP with NMR relaxation technology, and NMR reflects the geometric characteristics and connectivity of pores by detecting the relaxation time of hydrogen atoms in the material^36,37^. By combining these two techniques, a more comprehensive understanding of the evolution law of pore structure in cement-based materials during sulfate attack can be achieved, thereby providing a scientific basis for evaluating their strength degradation and durability. In summary, few studies have concurrently employed NMR and MIP techniques to investigate the pore changes in cementitious materials under Sulfate attack. Therefore, this research systematically explores the pore distribution characteristics and strength degradation mechanisms of cementitious materials subjected to Sulfate erosion through the application of MIP and NMR technologies.

This paper aims at investigating pore modifications occurring within cement paste, mortar, and concretized matrices subjected fully immersed into sulfate solutions whilst quantitatively analyzing resultant pore transformations.This study aims to mitigate salt-induced deterioration in cement-based materials, thereby extending service life and reducing disruptions caused by sulfate attack.Furthermore, it strives towards reducing waste generated through repetitive constructions necessitated owing damages inflicted onto existing infrastructures caused directly attributable back down salt-related erosions thereby contributing modestly towards sustainable development initiatives aimed ultimately lowering carbon dioxide emissions whilst promoting greener low-carbon methodologies throughout contemporary building practices.

## Materials and methods

### Raw materials

The cement selected is P·O 42.5R grade cement from Xinjiang Tianshan Cement Co., Ltd., which belongs to CEM II/A type. The chemical composition is shown in Table 1. The mixing water employed is tap water sourced from Kashgar; the superplasticizer used is a liquid polycarboxylic acid-based superplasticizer. The fine aggregate consists of river sand with a fineness modulus of 2.51, classified as medium sand, while the coarse aggregate comprises gravel with particle sizes ranging from 5 to 20 mm and an apparent density of 2650 kg/m³.

**Table 1:** Chemical composition of cement and clinker composition.

Ingredient	Mass fraction/%										
	SiO_2_	Al_2_O_3_	Fe_2_O_3_	CaO	MgO	SO_3_	K_2_O	Na_2_O	F-CaO	loss	Others
	*n*	*P*	C_3_S	C_2_S	C_3_A	C_4_AF	*R*_2_O	*R*_2_O	*R*_2_O	KH	-
Cement	23.21	4.86	3.81	55.63	1.39	2.45	0.81	0.25	0.78	3.69	3.12

### Mix proportion

In this experiment, three types of cement-based materials—namely cement paste, mortar, and concrete—were systematically designed. Maintaining a consistent water-cement ratio, a specific proportion of sand was incorporated into the cement paste to produce mortar, followed by the addition of a certain amount of stone to the mortar to yield concrete. This approach aimed to investigate the variations in different properties of these three cement-based materials post-erosion. The mix design adhered to the guidelines outlined in ‘Masonry Mortar Mix Ratio Design Regulations’ (JGJ/T 98–2011 of China) and ‘Ordinary Concrete Mix Ratio Design Regulations’ (JGJ55-2011 of China). The slump for concrete was controlled within the range of 150–180 mm. The mixing ratios for these cement-based materials are detailed in Table 2.

**Table 2:** Cement-based material mix ratio.

Number	Water-binder ratio	Cement-based materials amount of each material/(kg·m^−3^)				
		Water	Cement	Sand	Stone	Superplasticizer
P	0.35	508	1451	0	0	2.9
M	0.35	219	627	1254	0	1.2
C	0.35	156	448	896	896	0.9

Note: P, M and C are respectively cement paste, mortar and concrete.

### Test method

#### Sulfate attack regime

**Fig. 1:**
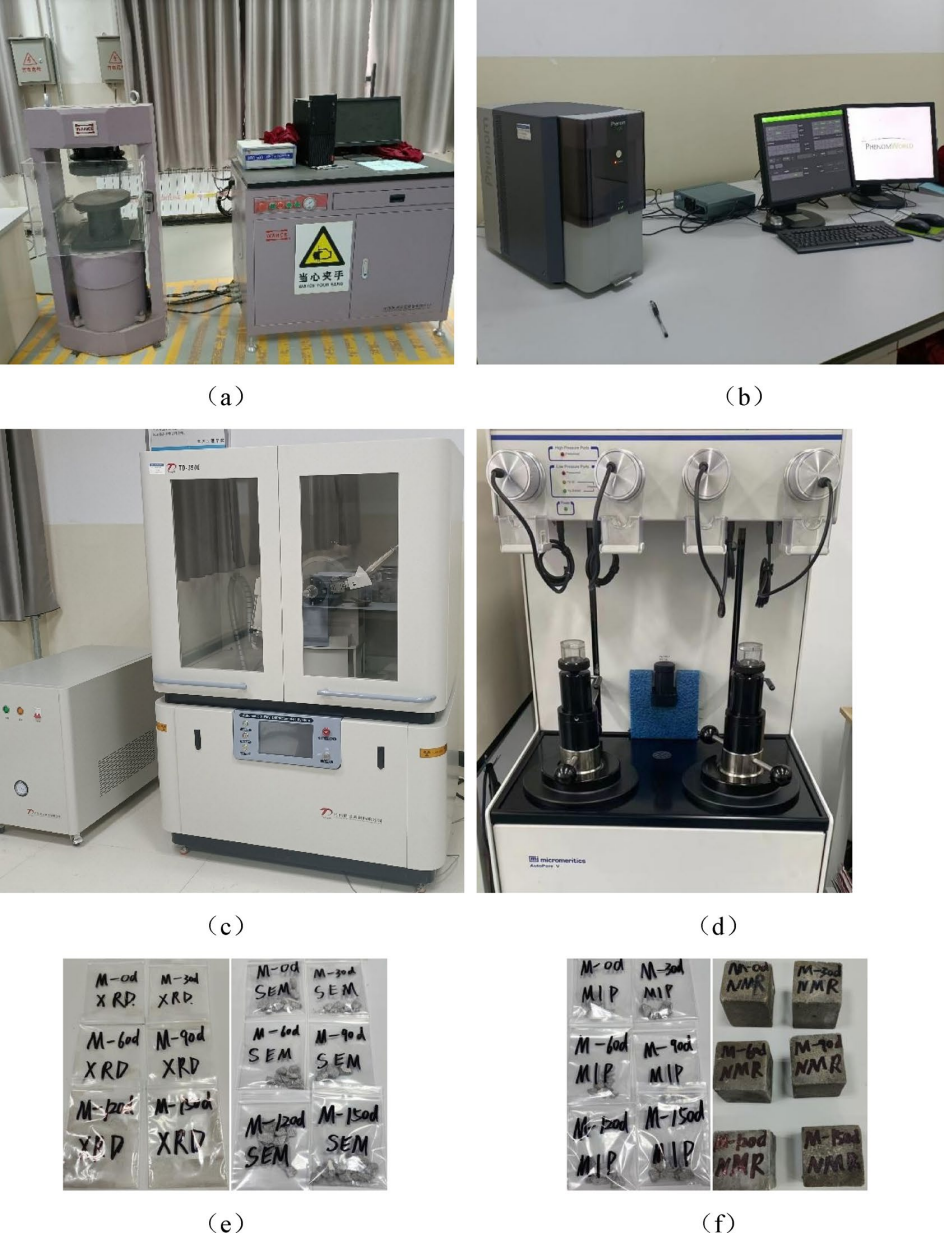
Test instruments (**a**) YAR-2000 Microcomputer controlled electro-hydraulic servo pressure testing machine; (**b**) Scanning electron microscopy; (**c**) X-ray diffraction; (**d**) Mercury Intrusion Porosimetry; (**e**) XRD and SEM testing of samples; (**e**) MIP and NMR testing of samples.

In this experiment, cement paste, mortar, and concrete cube samples with dimensions of 100 mm × 100 mm × 100 mm were prepared. Following a standard curing period of 28 days, the samples were placed in an oven at 80 °C for drying over a duration of 48 h. Once the specimens cooled down, they were immersed in a 5% Na_2_SO_4_ solution in a full immersion manner. To ensure the stability of the concentration of the corrosive solution, the container was sealed with cling film. Considering the dry climate of the experimental area, the concentration of the sodium sulfate solution was calibrated and tested weekly, and the corrosive solution was replaced every 30 days. At 30 days, 60 days, 90 days, 120 days and 150 days of immersion, the specimens were placed in an oven at 40℃ for 24 h^38,39^.The mass changes and compressive strengths of cement-based material specimens across each group at various ages were recorded; the average values from three test blocks per group were taken as final results.The press is shown in Fig. 1(a).

#### SEM analysis

Scanning Electron Microscopy (SEM) analysis was performed using a Phenom PROX machine to examine eroded specimens at different ages, As shown in Fig. 1(b). Specimens that had been eroded were fractured into flat sheets with diameters less than 5 mm and thicknesses approximately between 2 and 3 mm before being placed into a container filled with anhydrous ethanol to halt hydration processes; these sheets were subsequently baked in an oven set to maintain a temperature of 40 °C for a duration of twenty-four hours. The dried sheets were then positioned on a loading platform and transferred to a vacuum gold sputtering apparatus for gold coating treatment prior to observation under electron microscopy equipment.As shown in Fig. 1(e), it is the test sample.

#### X-ray diffraction analysis

An X-ray Diffraction (XRD) analysis was conducted utilizing instrumentation produced by Dandong Tongda Technology Co., Ltd., aimed at determining the phase composition within samples subjected to erosion over varying durations, as shown in Fig. 1(c). Core sampling equipment was employed to extract specimens from specimens eroded to different ages, after which the samples were placed in anhydrous ethanol to terminate hydration. Finally, the samples were ground in a mortar until the powder felt fine to the touch with no granular sensation. They were then sieved using a 45 μm mesh to obtain powder with a diameter less than 45 μm, which was placed in the instrument for testing. The scanning range was 5–90°, employing continuous scanning with a step width of 0.02°.As shown in Fig. 1(e), it is the test sample.

#### Mercury injection analysis

Mercury Intrusion Porosimetry (MIP) testing was executed utilizing the AutoPore IV9500 mercury intrusion porosimeter manufactured by McMurtry Equipment Co., Ltd.,as shown in Fig. 1(d). Take a sample with a diameter less than 10 mm and a thickness of approximately 5 mm from the reserved specimen. Immerse the sample in anhydrous ethanol for 48 ± 0.5 h to terminate hydration. Test the pore changes in specimens at different erosion ages. High-pressure testing capability up to 31,000 psi, contact angle of 130°, mercury surface tension of 480 mN/m, pore size measurement range from 0.003 to 360 μm.The MIP method is an invasive measurement approach. Its fundamental principle is that non-wetting mercury liquid can only invade the pores of materials under applied pressure. The measurement range of MIP is quite wide, generally from 3 nm to 1000 μm. It is particularly adept at characterizing mesopores to macropores (50–200 nm), and can fully cover the pore range below 200 nm that is the focus of this study. However, it has certain destructive aspects. After testing, the samples will be contaminated by mercury, and the high pressure may also damage the sample structure.As shown in Fig. 1(f), it is the test sample.

#### Low-field nuclear magnetic analysis

Nuclear Magnetic Resonance (NMR) Testing: The MesoMR12-060 H-I high-precision magnetic resonance concrete microanalysis instrument produced by Suzhou NuoMai Analytical Instrument Co., Ltd. in China was used. Specimens measuring 40 mm×40 mm×4 mm were prepared, standard-cured for 90 days, and then placed in a vacuum water-retaining machine. After water retention was completed. The pore changes of specimens with different water-cement ratios were tested, with a pore size measurement range of 0.00002 to 200 μm.As shown in Fig. 1(f), it is the test sample.NMR is a non-invasive measurement method. Its basic principle is to utilize the relaxation characteristics of hydrogen nuclei in pore fluids (usually water or oil) in a magnetic field. The relaxation time of the fluid in different-sized pores varies, and by detecting the relaxation signals, information such as pore structure, fluid distribution, and permeability can be inversely calculated. It can perform in-situ monitoring quickly and non-destructively. However, it also has certain limitations. The results of pore size distribution depend on the conversion model between relaxation time and pore size.This study adopts the conversion formula of relaxation time and porosity^40^, as shown in Eq. (1).

1 $$\:{r}_{pore}=\frac{\rho\:\times\:{F}_{S}}{(\frac{1}{{T}_{2}}-\frac{1}{{T}_{2B}})}\:$$

In the formula, *r*_*pore*_ represents the pore diameter, measured in nanometers; *ρ* represents the surface relaxation rate, typically ranging from 0.0005 to 0.0015 cm/s, and is set to 0.001 cm/s^41^; the pores are regarded as cylindrical bodies, and *F*_*S*_ is taken as 2; *T*_*2*_ is the total relaxation time for measurement, measured in seconds; *T*_*2B*_ is the transverse relaxation time of liquid water, usually set at 2s^42^.

## Results and analysis

### Mass change after sulfate attack

**Fig. 2:**
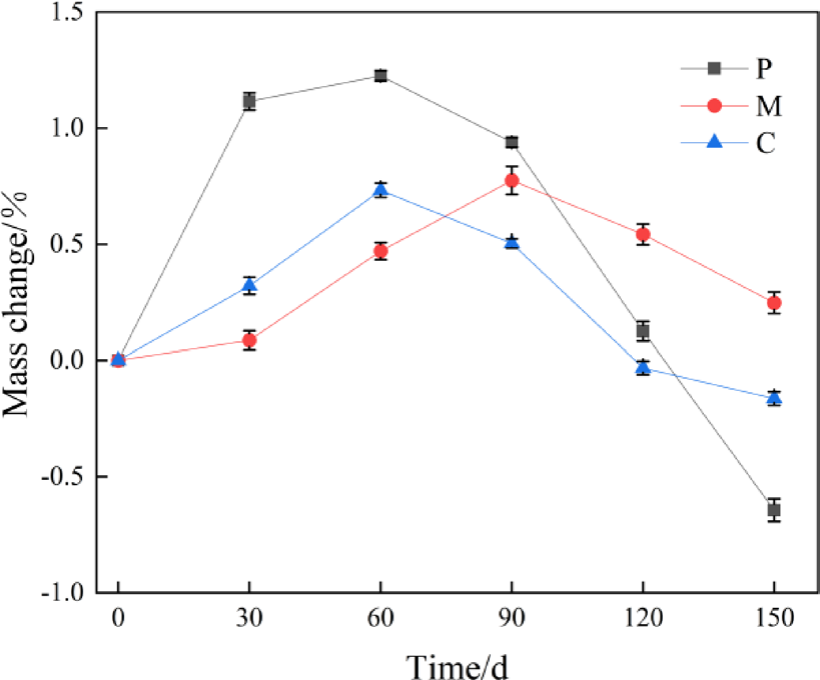
Mass loss map of cement-based material at 150 d of sulfate attack.

Figure 2 illustrates the changes in mass of cement-based materials subjected to sulfate attack over a period of 150 days. As depicted in the figure, the mass variations of cement paste, mortar, and concrete specimens generally exhibit an initial increase followed by a subsequent decrease. At 60 days of exposure, both cement paste and concrete reached their maximum mass change values, with increases of 1.22% and 0.73%, respectively. By day 150, the corresponding mass losses were recorded at 0.64% for cement paste and 0.16% for concrete. The mortar specimen experienced its peak mass change at day 90 with an increase of 0.77%, while at day 150 it showed a further increase of only 0.274%. This phenomenon can be attributed to the reaction between sulfate ions and gelling materials that produces erosion products such as gypsum and ettringite, thereby enhancing the specimen’s mass initially; however, as erosion progresses over time, these products accumulate leading to expansion and cracking within the specimen which ultimately results in material loss.

Following sulfate-induced erosion, the trend observed in mortar is analogous to that seen in mortar samples. The incorporation of fine aggregates stabilizes pore distribution within the mortar matrix; consequently, this leads to a reduced relative change in quality post-erosion with more gradual fluctuations compared to other specimens. Initially increasing with prolonged exposure time due to erosion effects on structural integrity—mortar begins experiencing reductions in quality thereafter as degradation sets in more prominently over time than previously noted trends suggest when coarse aggregates are included within concrete mixtures; thus resulting faster alterations during early stages owing primarily due interfacial transition zones formed from coarse aggregate addition—these zones represent weak links susceptible towards sulfate attacks^43,44^. Therefore relative changes regarding quality among concrete specimens are significantly influenced by these transitional interfaces where initial growth rates surpass those observed amongst mortars alongside earlier peaks occurring therein.”

### The change of compressive strength after sulfate attack

**Fig. 3:**
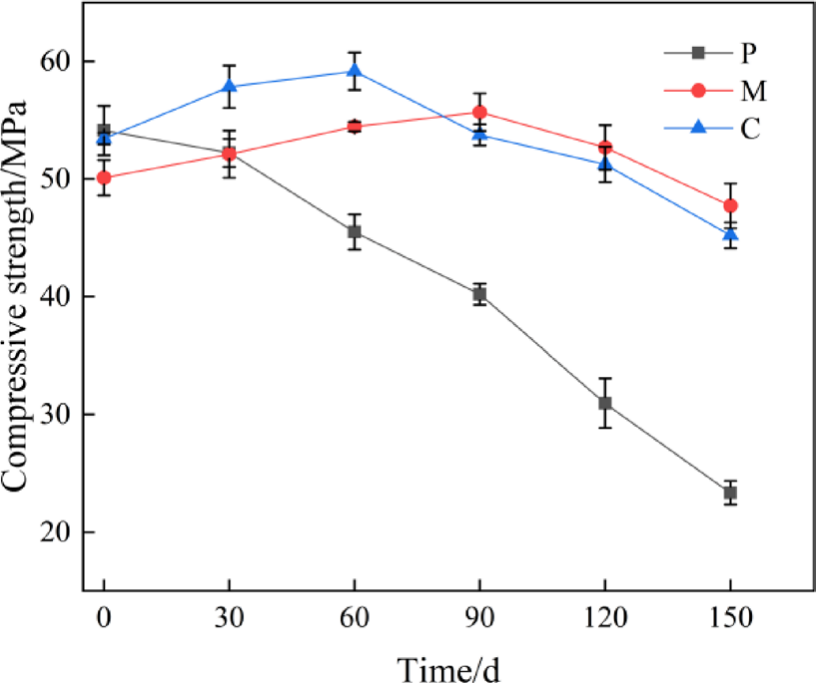
Changes in compressive strength of sulfate attack at 150d.

Figure 3 illustrates the variation in compressive strength over a period of 150 days under sulfate attack. The graph demonstrates that the compressive strength of the pure mortar specimens exhibited an overall decreasing trend, with an initial strength of 54.11 MPa before attack and reaching a minimum value of 23.34 MPa after 150 days of attack. Mortar and concrete specimens exhibited an overall trend of initially slow increase followed by decrease in compressive strength. Mortar reached its maximum compressive strength of 55.67 MPa at 90 days of erosion, decreasing to a minimum of 47.71 MPa at 150 days, representing a reduction of 14.29%. Concrete specimens exhibited peak compressive strength of 59.15 MPa at 60 days of erosion, declining to a minimum of 45.20 MPa at 150 days—a 19.34% reduction. This is attributable to the presence of an interfacial transition zone within the concrete. During the initial stages of Sulfate attack, this zone exhibits a certain strengthening effect on the material’s strength. However, as the duration of attack prolongs, Sulfates gradually permeate into the material’s interior, exerting a destructive influence that leads to a reduction in compressive strength^45–48^.Furthermore, the data indicates that the strength reduction in concrete specimens was greater than that in mortar specimens, suggesting that concrete is more significantly affected by Sulfate erosion. This is also related to the presence of a relatively weak interfacial transition zone within concrete, which is more susceptible to deterioration under prolonged erosion, consequently leading to a reduction in overall compressive performance.

During the corrosion process, Sulfate ions initially react with calcium hydroxide (a cement hydration product) within the cementitious matrix to form gypsum. This gypsum then reacts with aluminium-containing hydration products to produce aluminate cement. The resulting gypsum and aluminate cement continuously fill the internal pores of the cementitious matrix specimen, causing its strength to increase^49^. During this stage, the increase in compressive strength is closely related to changes in the pore structure of the cementitious material. In the later stages of erosion, expansive forces disrupt the pore structure of the cementitious material, leading to a decrease in its strength.

### XRD phase analysis

**Fig. 4:**
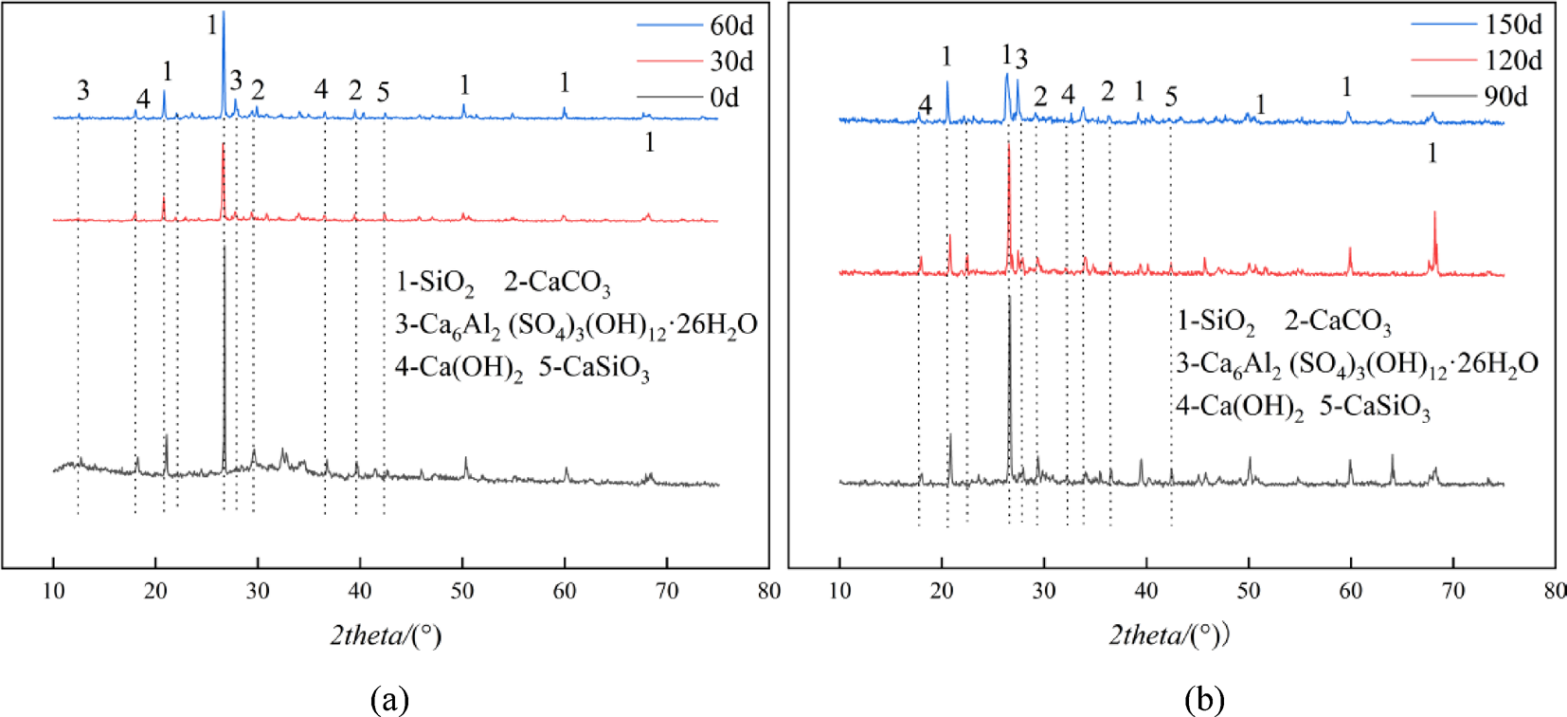
XRD image of mortar with 150 sulfate attack (**a**) 0 d, 30 d, 60 d; (**b**) 90 d, 120 d, 150d.

To understand the erosion products after sulfate attack, X-ray diffraction analysis was conducted on mortar specimens at 0 days, 30 days, 60 days, 90 days, 120 days, and 150 days of erosion. As shown in Fig. 4, at 0 days, the main phases were silica, calcium carbonate, calcium hydroxide, and calcium silicate, etc. After sulfate attack, the diffraction peak of calcium hydroxide remained, indicating that the hydration of cement had not stopped. At 30 days of erosion, ettringite was added as the main phase, suggesting that the sulfate attack was mainly due to the chemical reaction between sulfate ions and the hydration products of cement, resulting in the formation of ettringite and causing damage to the cement-based material^50^. With the continuous increase of the erosion age, the types of products formed during the erosion process did not change.

### Microscopic morphology analysis by scanning electron microscopy

**Fig. 5:**
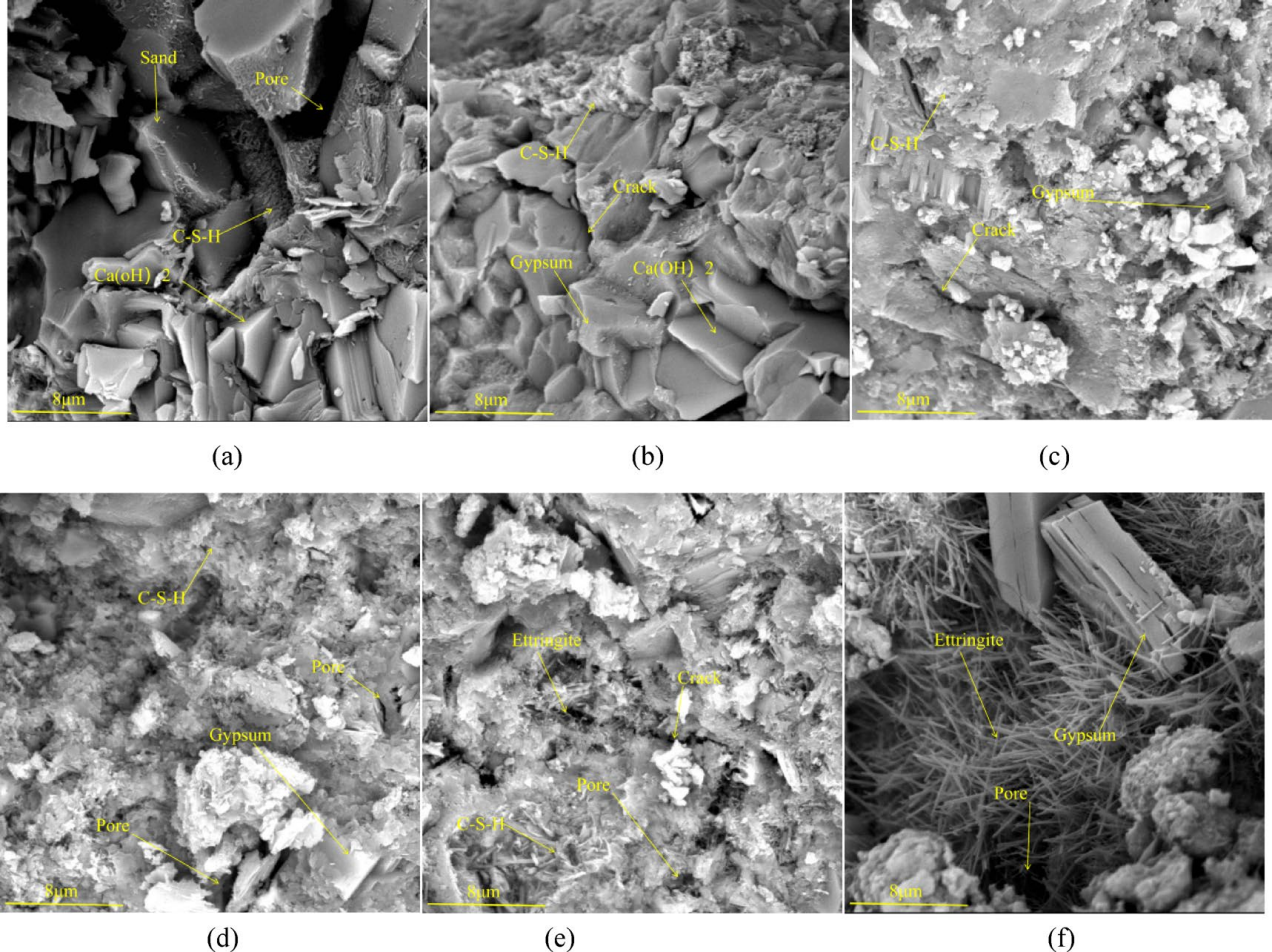
SEM image of mortar with 150 sulfate attack (**a**) 0 d; (**b**) 30 d; (**c**) 60 d; (**d**) 90 d; (**e**)120d; (**f**)150d.

Figure 5 shows the SEM images of mortar specimens subjected to sulfate attack at 0 d, 30 d, 60 d, 90 d, 120 d and 150d. It can be seen from the figure that the microstructure inside the specimens varies significantly at different attack ages. As shown in (a), the internal microstructure of the specimen at 0 d of attack is relatively smooth, indicating good cement hydration. As shown in (b), at 30 d of attack, the internal microstructure is different from the smooth surface of the unattacked specimen, with short columnar products generated inside and erosion products beginning to adhere to the surface. The pore diameter starts to decrease, indicating that the erosion products have been formed and are beginning to fill the pores inside the specimen. As shown in (c), at 60 d of attack, there are a few pores and microcracks, indicating some damage. This is due to the increasing amount of erosion products, which causes microcracks in the specimen. As shown in (d), at 90 d of attack, the internal pore surface has become uneven, with gypsum and ettringite covering the originally smooth surface and further filling the pores. At this point, the strength of the mortar reaches its peak. The filling effect of the internal products on the pores is greater than the effect of the decomposition of the cementitious materials on the strength, so the macroscopic manifestation is an increase in compressive strength and mass change. As shown in (e), at 120 d of attack, a small amount of ettringite begins to form inside the specimen. As shown in (f), at 150 d of attack, the internal morphology of the specimen is significantly different from the initial state. Due to the continuous accumulation of erosion products, a large number of needle-like ettringite products appear inside, causing the internal morphology to become honeycomb-like. The macroscopic manifestation is a decrease in compressive strength and mass change.

### Pore changes after sulfate attack based on mercury injection technique

**Fig. 6:**
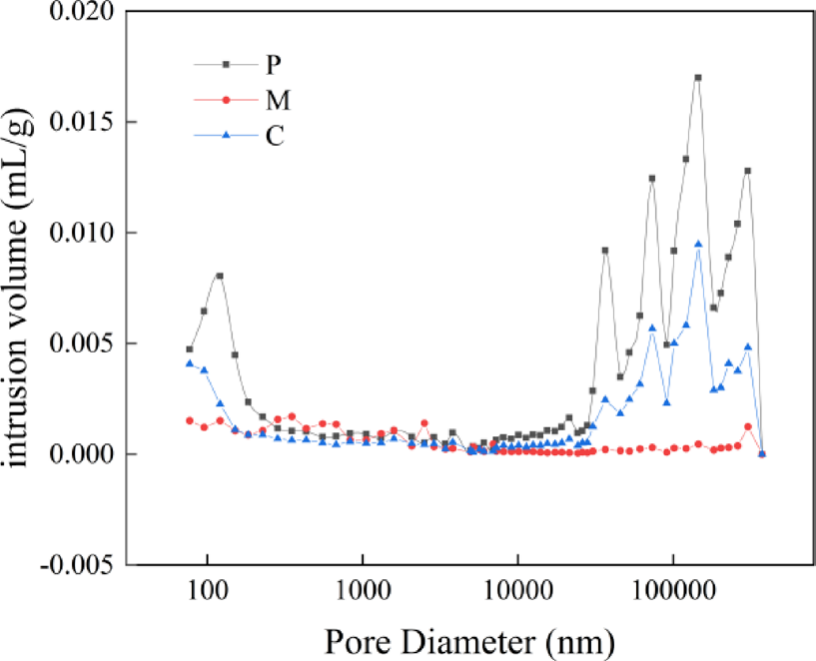
The pore size distribution curves of the cement paste, mortar and concrete after 28 days of curing.

Figure 6 illustrates the pore size distribution curve of cleancement paste, mortar, and concrete after 28 days of curing. It can be seen from Fig. 5; Table 3 that, under a constant water-cement ratio, the cement cleancement paste exhibits the largest pore distribution and porosity at 26.38%. Upon incorporating sand, there is a significant reduction in pores smaller than 100 nm and larger than 10,000 nm; consequently, the pore size distribution becomes more uniform and porosity decreases to 13.58%. With the addition of coarse aggregate, heterogeneity within the cement-based material increases, leading to an increase in harmful pores exceeding 10,000 nm compared to mortar; thus raising porosity to 20.06%.

**Fig. 7:**
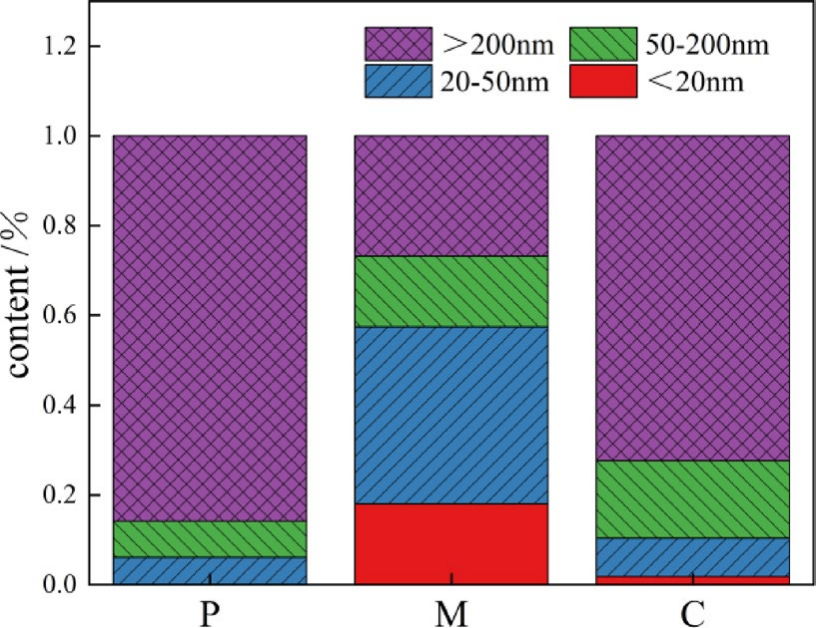
The stacking chart of pore changes of net cement paste, mortar and concrete after curing for 28 days.

Figure 7 presents a diagram depicting changes in pore structure and accumulation for cement paste, mortar, and concrete over a curing period of 28 days. According to Academician Wu Zhongwei^51,52^, internal pore diameters in cement-based materials are classified into several categories: Level One: harmless pores (0 nm − 20 nm); Level Two: less damaging pores (20 nm − 50 nm); Level Three: harmful pores (50 nm −200 nm); Level Four: multiple holes (greater than 200nm). As illustrated in the figure, proportions of harmless holes, less harmful holes, harmful holes and highly harmful pores in cement paste are recorded as follows: 0% for harmless holes; 6.06% for less harmful; 8.07% for harmful; and an overwhelming majority of 85.86% for highly harmful pores—indicating a predominance of detrimental structures due to fine cement particles being susceptible to environmental temperature fluctuations which lead them to shrink or expand.In contrast with mortar’s composition where proportions stand at: harmless holes (18.02%), less harmful (39.48%), harmful (15.66%) and multi-harmful (26.82%), it is evident that mixing sand significantly enhances both harmless hole ratios while reducing those categorized as multi-harmful due primarily to volume stability provided by fine aggregates which mitigate overall shrinkage within cement-based materials—resulting in predominant volumes found within the diameter range of approximately ten-to-one hundred nanometers.For concrete samples analyzed post-coarse aggregate incorporation reveal proportions as follows: harmless holes at just above 2% (1.82%), slightly damaged at eight point five 4% (8.54%), harmfully affected at seventeen point twenty-nine%(17.29%)and predominantly multiharmed structures comprising seventy-two point thirty-four%(72.34%). This shift reflects how stone filling complicates pore pathways while amplifying larger voids particularly around interface transition zones.”

**Fig. 8:**
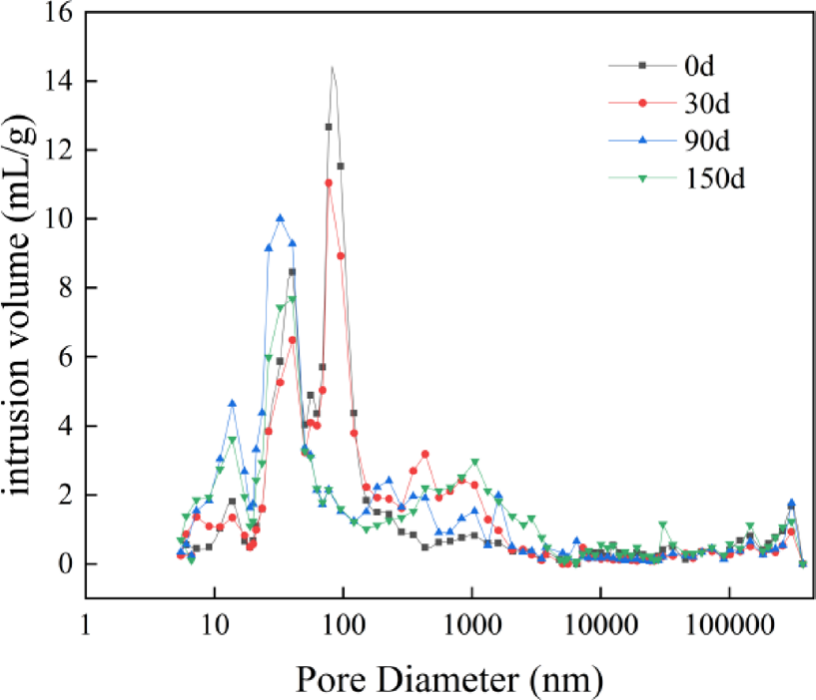
Pore size distribution curve of 0 d, 30 d, 90 d and 150 d mortar subjected to sulfate attack.

Figure 8 shows the pore size distribution curves of mortar for sulfate attack at 0 d, 30 d, 90 d, and 150d. From the figure, it can be seen that sulfate attack has a significant impact on the pore size range of 10 nm to 10,000 nm in the mortar. At 30 d of sulfate attack, the mercury penetration in the 10–100 nm range and the range greater than 10,000 nm is less than that of 0 d, while the mercury penetration in the 100 nm-10,000 nm range is greater than that of 0d. The porosity rate decreases from 13.58% at 0 d to 12.57% at 30 d of sulfate attack. This is because in the early stage of sulfate attack, the sulfate will react with the hydraulic binder materials to generate expansive products to fill the pores, resulting in a decrease in porosity rate. At 90 d of sulfate attack, the mercury penetration in the 0–75 nm range and the range greater than 100,000 nm is less than that of 30 d, while the mercury penetration in the 75 nm-100,000 nm range is greater than that of 30d. The porosity rate increases from 12.57% at 30 d to 14.17% at 90 d of sulfate attack. This is because in the middle and late stages of sulfate attack, as the reaction proceeds and the products accumulate, the generated expansive force increases, and when the expansive force exceeds the tensile strength of the pore limit, the internal structure of the specimen will be destroyed, causing the pores to enlarge. At 150 d of sulfate attack, the mercury penetration in the range greater than 10,000 nm reaches the maximum, and the porosity rate increases to 18.27%. This is because in the late stage of sulfate attack, the sulfate products will continue to accumulate, and the expansive force will become.

**Fig. 9:**
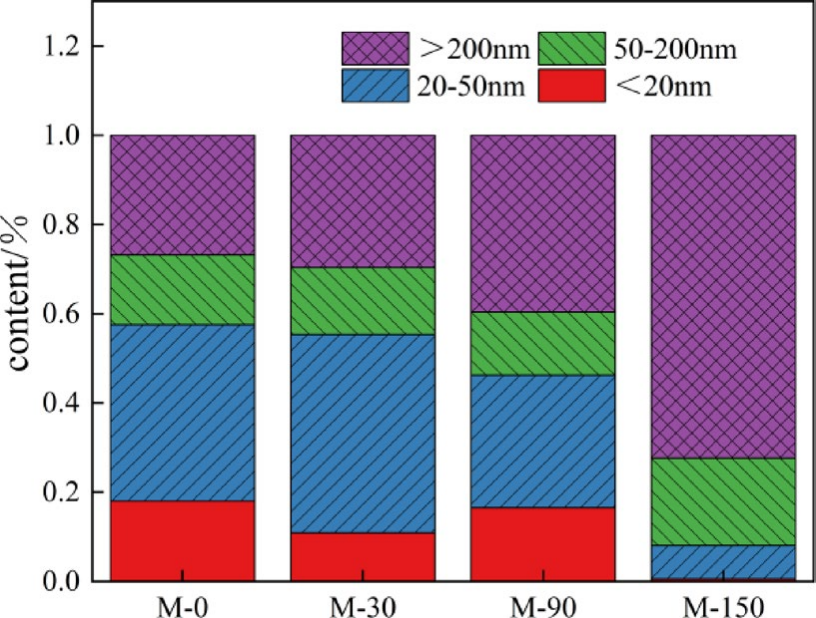
Pore change and accumulation map of 0 d, 30 d, 90 d and 150 d mortar subjected to sulfate attack.

Figure 9 illustrates the changes in pore structure and accumulation patterns of mortar subjected to sulfate erosion over periods of 0, 30, 90, and 150 days. As evident from both Fig. 9; Table 3, the proportion of harmful pores exhibits a trend of initially decreasing followed by an increase with increasing attack duration. In uneroded mortar, the proportions of harmless pores, slightly damaged pores, harmful pores, and multiple damage pores were recorded at 18.02%, 39.48%, 15.66%, and 26.82%, respectively. After a period of 30 days under erosive conditions, these proportions shifted to 10.86% for harmless pores, 44.43% for slightly damaged ones, while harmful and multi-harmful pore percentages were noted at 15.07% and 29.62%. This change reflects a decrease in both harmless and harmful pore ratios alongside an increase in less harmful and multi-harmful categories due to expansion products generated by sulfate attack filling previously harmful voids—effectively reducing their diameter into less damaging classifications. Following an additional erosion period leading up to day-90 results showed proportions at: harmless (16.49%), slightly damaged (29.72%), harmful (14.18%), with multiple damage increasing significantly to reach a total of (39.60%). Here again we observe a decline in both slight damage and harm categories while there is an uptick in both harmlessness as well as multiple damages observed post-erosion phase extending through day-150 where final measurements indicated: harmless holes at just (0 0.59%), slightly harmed at (7 0.41%), with significant rises seen within harmful holes reaching (19 0.59%) along with more severely affected holes escalating dramatically to account for (72 0.40%). Notably here too was evident reduction among benign or mildly impacted voids contrasted against notable increases within detrimental classifications; this phenomenon can be attributed primarily towards late-stage erosional processes wherein internal porosity becomes filled by resultant products yet continues generating further expansive forces which exacerbate crack propagation throughout mortar matrices thereby amplifying overall porosity levels^53^.

**Table 3:** MIP tests aperture characteristics parameters.

Specimen	Porosity/%	<20 nm	20 nm ~ 50 nm	50 nm ~ 200 nm	>200 nm
P	26.38	0.00%	6.06%	8.07%	85.86%
M	13.58	18.02%	39.49%	15.66%	26.83%
C	20.06	1.82%	8.54%	17.30%	72.34%
M-30	12.57	7.87%	21.44%	41.08%	29.62%
M-90	14.17	16.49%	29.73%	14.18%	39.60%
M-150	18.27	0.59%	7.41%	19.59%	72.40%

### Pore changes after sulfate attack based on low-field nuclear magnetic resonance technology

The transverse relaxation time T2 spectrum of nuclear magnetic resonance reflects the distribution of pore sizes in cement-based materials. The shorter the transverse relaxation time T2, the smaller the pore radius; the longer the transverse relaxation time T2, the larger the pore radius. The position of each peak in the T2 spectrum distribution curve is related to the pore size, and the size of the integral area of each peak reflects the change in the number of internal pores in cement-based materials^54^.

**Fig. 10:**
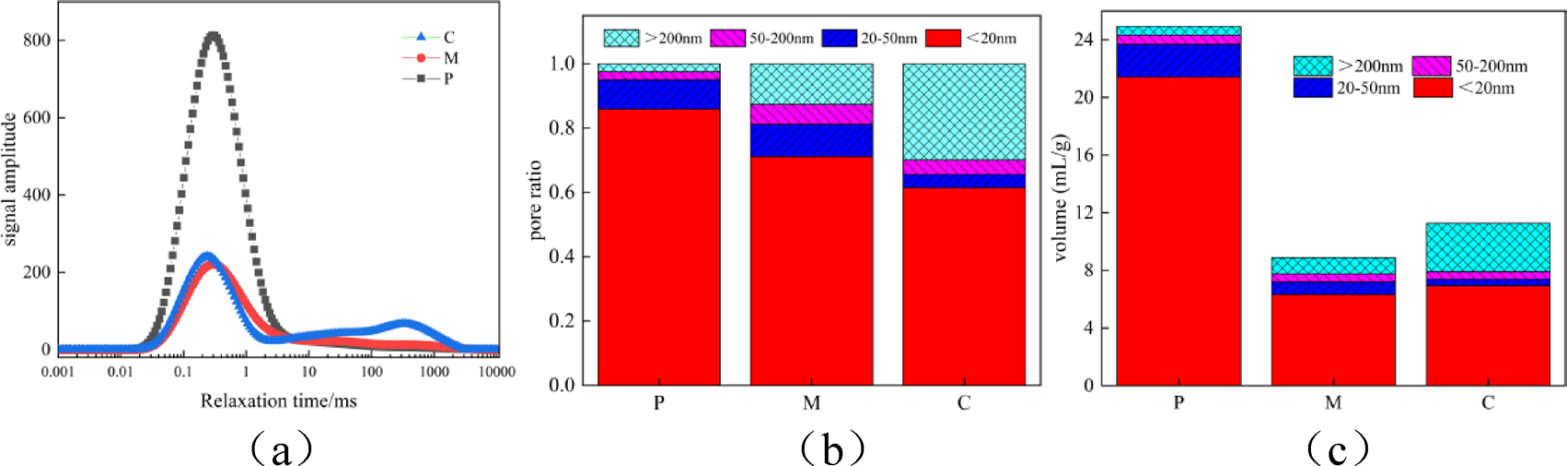
The P, M and C magnetic resonance imaging analysis diagrams after 28 days of maintenance; (**a**) PMCT2 spectrum analysis curve (**b**) Pore distribution (**c**) pore change.

Figure 10 shows the nuclear magnetic resonance analysis diagrams of P, M, and C after 28 days of curing. From Figure (a), it can be seen that the nuclear magnetic resonance T2 spectra of P and M mainly present a “main peak” structure. The main peaks are all distributed at shorter transverse relaxation times, and the signal intensity shows P > M, indicating that the number of small pores inside P and M accounts for a larger proportion, with only a small part being large pores. The transverse relaxation time T2 of P is between 0.017 and 3217.642 ms, and that of M is between 0.022 and 4824.108 ms. The nuclear magnetic resonance T2 spectrum of C mainly presents a “main and secondary peak” structure. The main peaks are all distributed at shorter transverse relaxation times, and their signal intensity and the integral area under the distribution curve are significantly larger than those of the secondary peaks, indicating that the number of small pores inside the concrete accounts for a larger proportion, with only a small part being large pores. The transverse relaxation time T2 of C is between 0.02 and 2967.3 ms. It can be clearly seen from Figure (a) that the porosity of the cement paste specimens is relatively large, and with the addition of sand and gravel, the internal bonding force of the matrix is increased, and the fine and coarse aggregates are improved, thereby improving the pore structure, and the number of pores in the cement-based materials gradually decreases, and the porosity gradually decreases.

As shown in Fig. 10(b) and (c), the pore change accumulation diagrams of the cement paste, mortar, and concrete are presented. Combining Table 4 with the statistical description of the T2 spectrum distribution curve from nuclear magnetic resonance, the proportions of P, M, and C pore types are characterised. It can be seen from the figure that the porosity of P is 24.93%, with the proportions of harmless pores, slightly harmful pores, harmful pores, and highly harmful pores being 85.86%, 9.16%, 2.5%, and 2.46% respectively; the porosity of M is 8.88%, with the proportions of harmless pores, slightly harmful pores, harmful pores, and highly harmful pores being 71.14%, 10%, 6.23%, and 12.62% respectively; the porosity of C is 11%, with the proportions of harmless pores, slightly harmful pores, harmful pores, and highly harmful pores being 61.5%, 4.12%, 4.49%, and 29.87% respectively. For the cement paste, mortar, and concrete specimens, the porosity continuously decreases with the addition of sand, and increases with the addition of sand and gravel. The proportions of harmless pores, slightly harmful pores, and harmful pores decrease, while the proportion of highly harmful pores increases.

Analysis of the reasons: The addition of sand and gravel improves the pore structure of the material to a certain extent. As aggregates, sand and gravel can fill some pores, reducing the formation of harmful and highly harmful pores, thereby increasing the density and overall performance of the material. However, after adding gravel to the mortar, although the internal bonding force of the matrix is further enhanced, more interface transition zones are introduced, which are often weak and prone to become concentrated areas of pores and micro-cracks, resulting in an increase in porosity, especially a significant increase in the proportion of highly harmful pores. In addition, the pore structure of the gravel itself may also affect the overall porosity.

**Fig. 11:**
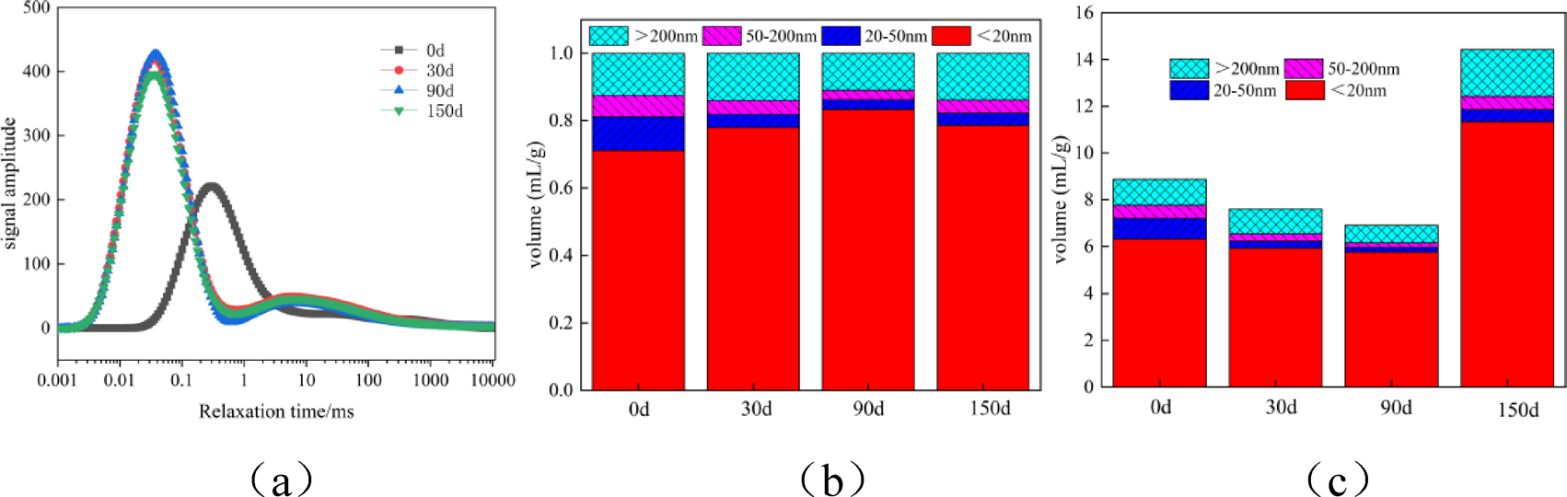
Nuclear magnetic resonance analysis diagram of mortar specimens after sulfate attack; (**a**) Analysis curves of the T2 spectra for different erosion ages of mortar(**b**) Pore distribution (**c**) pore change.

Figure 11 shows the NMR analysis of M after 150 days of sulfate attack. As can be seen from Figure (a), the NMR T2 spectrum of M before erosion mainly presents a “main peak” structure. The main peaks are all distributed at the shorter transverse relaxation time, with T2 ranging from 0.022 to 4824.108 ms, indicating that the number of small pores inside M accounts for a large proportion, while only a small part are large pores. After erosion, the NMR T2 spectrum of M mainly presents a “main and secondary peak” structure. The main peaks are all distributed at the shorter transverse relaxation time, and the signal intensity and the area under the distribution curve of the main peaks are significantly greater than those of the secondary peaks, indicating that the pore structure inside the mortar has undergone significant changes after sulfate attack. The transverse relaxation time T2 of M after erosion ranges from 0.002 to 10,000 ms.

As shown in Figs. 11(b) and (c), they are the pore change accumulation diagrams of the mortar after sulfate attack. Combining Table 4 with the statistical description of the distribution curve of the T2 spectrum from nuclear magnetic resonance, the proportion of mortar pores before and after erosion is characterised. It can be seen from the figure that the porosity of M before erosion is 8.88%, and the proportions of harmless pores, slightly harmful pores, harmful pores, and highly harmful pores are 71.14%, 10%, 6.23%, and 12.62% respectively. After 30 days of erosion, the porosity is 7.60%, and the proportions of harmless pores, slightly harmful pores, harmful pores, and highly harmful pores are 77.93%, 3.91%, 4.13%, and 14% respectively. The proportions of harmless pores and highly harmful pores increase, while the proportions of slightly harmful pores and harmful pores decrease. After 90 days of erosion, the porosity is 6.91%, and the proportions of harmless pores, slightly harmful pores, harmful pores, and highly harmful pores are 83.35%, 2.85%, 2.85%, and 10.93% respectively. The proportion of harmless pores increases, while the proportions of slightly harmful pores, harmful pores, and highly harmful pores decrease. After 150 days of erosion, the porosity is 14.42%, and the proportions of harmless pores, slightly harmful pores, harmful pores, and highly harmful pores are 78.58%, 3.62%, 3.93%, and 13.86% respectively. The proportions of harmless pores and slightly harmful pores decrease, while the proportions of harmful pores and highly harmful pores increase. This is because, in the initial stage of erosion, sulfate will react chemically with the internal substances of the cement-based material to form gypsum and other substances, filling the internal pores of the specimen, reducing the porosity and the proportions of harmful pores and highly harmful pores. In the later stage of erosion, as the chemical reaction continues, the products increase. When the volume of the products reaches a certain amount, it will cause the specimen to crack, increasing the proportions of harmful pores and highly harmful pores.

**Table 4:** NMR tests aperture characteristics parameters.

Specimen	Porosity/%	<20 nm	20 nm ~ 50 nm	50 nm ~ 200 nm	>200 nm
P	24.93	85.87%	9.17%	2.50%	2.46%
M	8.88	71.14%	10.00%	6.24%	12.62%
C	11	61.50%	4.13%	4.49%	29.88%
M-30	7.60	77.94%	3.92%	4.13%	14.01%
M-90	6.91	83.36%	2.86%	2.86%	10.93%
M-150	14.42	78.59%	3.62%	3.94%	13.86%

**Fig. 12:**
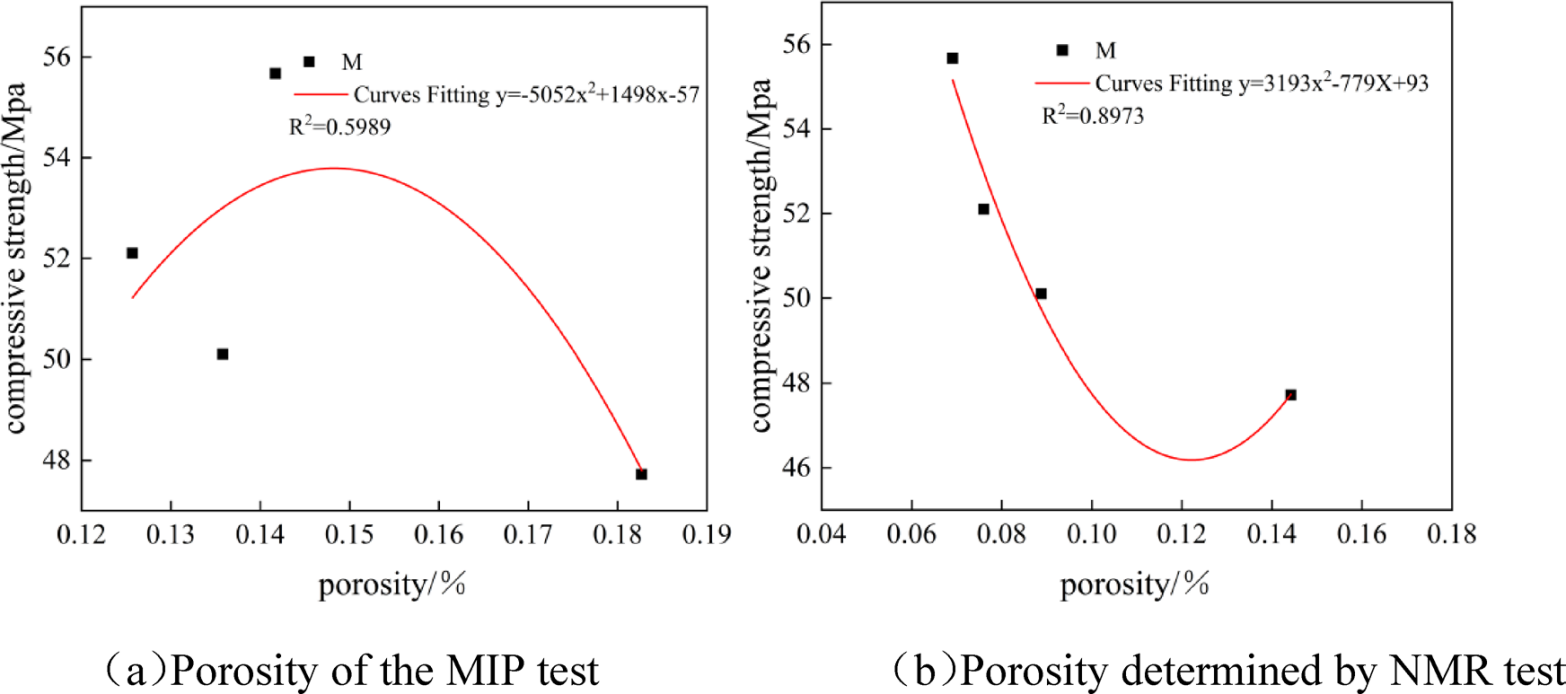
Fitting curves of porosity and compressive strength for mortars. (**a**) Porosity of the MIP test, (**b**) Porosity determined by NMR test.

Figure 12 shows the fitting curves of the porosity and compressive strength of mortar specimens at different sulfate attack ages. It can be seen from the figure that the compressive strength of mortar decreases continuously with the increase of porosity. Figure (a) is the fitting curve of the porosity and compressive strength of mortar specimens tested by MIP, with a relatively small correlation coefficient of 0.5989, and the test results are relatively scattered. Figure (b) is the fitting curve of the porosity and compressive strength of mortar specimens tested by NMR, with a relatively small correlation coefficient of 0.8973, and the test results have a relatively good correlation. There is a certain difference in the porosity measured by the two methods of NMR and MIP, mainly because the samples tested by pressure mercury are relatively small and do not have sufficient representativeness.

The pore changes of mortar specimens were analyzed by combining the pressure mercury method and nuclear magnetic resonance technology. It was found that there were certain differences in the porosity measured by the two methods, but the changing trends were consistent. This was mainly due to the different sample sizes tested by the two methods, which led to minor differences in the measurement results. The pressure mercury method is usually suitable for smaller volume samples and can more accurately reflect the local pore structure, while nuclear magnetic resonance technology is suitable for larger volume samples and can provide more comprehensive pore distribution information. Despite the differences, both methods indicated that with the increase of erosion age, the porosity of cement-based materials first decreased and then increased. By comprehensively analyzing the test results of the two methods, the influence mechanism of pore structure on the performance of cement-based materials can be better understood, providing important references for optimizing material ratios and improving material performance.

## Conclusion

This study focuses on cement-based materials, examining the impact of sulfate attack on the properties of cement paste, mortar, and concrete. The following conclusions can be drawn:

(1) The mass changes of cement paste, mortar and concrete increase first and then decrease with the increase of sulfate attack age. The mass of cement paste and concrete reaches the maximum at 60 days of attack, increasing by 1.22% and 0.73% respectively, and then decreases by 0.64% and 0.16% respectively at 150 days. While the mass of mortar reaches the maximum at 90 days of attack, increasing by 0.77%, and then increases by 0.274% at 150 days.

(2) The compressive strength of the cement paste decreases with the increase of the erosion age, reaching a minimum value of 23.34 MPa, with a reduction rate of 56.86%. The compressive strength of the mortar and concrete first increases and then decreases. At 150 days of erosion, the compressive strength of the mortar decreases by 14.29%, and the compressive strength of the concrete decreases by 19.34%. This indicates that the optimized mix design with adjusted aggregate content can alleviate the strength changes caused by sulfate.

(3) For specimens of cement paste, mortar and concrete, the porosity is in the order of neat paste > concrete > mortar before sulfate attack. After 30 days of sulfate attack, the porosity of mortar first decreases and then increases. Based on mercury intrusion porosimetry, the porosity of mortar decreases by 1.01% after 30 days of sulfate attack and increases by 5.7% after 150 days. Based on low-field nuclear magnetic resonance technology, the porosity of mortar decreases by 1.28% after 30 days of sulfate attack and increases by 5.54% after 150 days.

(4) The pore changes of mortar were analyzed by combining MIP and NMR techniques. It was found that the porosities measured by the two methods were different because MIP testing could only measure the “throat” or “entrance” size of the pore network and could not detect closed pores, dead-end pores, etc. However, the overall change trends were consistent.

## Supplementary Information

Below is the link to the electronic supplementary material.

Supplementary Material 1

Supplementary Material 2

Supplementary Material 3

Supplementary Material 4

## Data Availability

All data are available upon request from the corresponding author.
