# Supplementary material for: Research on the pore distribution characteristics and strength degradation of cement-based materials under sulfate attack
Source: Sci Rep. 2025 Dec 7;16:1460. doi: 10.1038/s41598-025-31233-5 (PMC12796256; doi:10.1038/s41598-025-31233-5)
Supplement: Supplementary file 1 — Supplementary Material 1 [file 41598_2025_31233_MOESM1_ESM.pdf]

--	0-20	
	0	0.01274
	30	0.00473
	90	0.0157
	150	9.80E-04

	0-20
P	0
M	0.01274
C	0.00184

Pore Diameter	intrusion volume	Cumulative Volum	Pore Diameter	intrusion volume
nm	mL/g	mL/g	nm	mL/g
1	孔容	累计体积	2	孔容
369796.05	0	1.19E-30	370375.6	0
302966.05	0.01182	0.01182	303194.475	0.01039
259303.5	0.00935	0.02117	258513.175	0.00797
226365.225	0.00831	0.02948	225947.475	0.00722
201667.4875	0.00537	0.03484	201591.7625	0.00673
181209.6625	0.00392	0.03876	180988.525	0.00463
144676.775	0.00161	0.05488	144612.8	0.0068
120682.475	0.00638	0.06126	120626.24	0.00661
100833.7438	0.00555	0.06681	100782.2438	0.0054
90340.8	0.00265	0.06946	90274.94375	0.00264
73016.74375	0.00464	0.0741	73077.18125	0.00489
60713.38125	0.00254	0.07664	60736.125	0.00264
52035.80313	0.0017	0.07833	52033.675	0.00179
45506.29063	0.00122	0.07956	45516.21875	0.00121
36358.09375	0.00172	0.08128	36364.98438	0.00165
30294.48	0.0012	0.08248	30282.47188	9.93E-04
27938.70938	4.53E-04	0.08293	27953.36	4.12E-04
25942.08906	4.08E-04	0.08334	25935.61875	3.15E-04
24208.4	3.85E-04	0.08372	24208.39375	2.91E-04
21361.375	7.02E-04	0.08442	21363.19375	5.09E-04
19109.44375	4.75E-04	0.0849	19106.47656	4.12E-04
17289.70781	5.21E-04	0.08542	17294.49375	3.87E-04
15448.325	4.98E-04	0.08592	15451.09531	3.63E-04
13925.30625	4.53E-04	0.08637	13965.60781	3.39E-04
12490.72109	5.21E-04	0.08689	12490.06016	3.87E-04
11320.09609	4.53E-04	0.08734	11320.37813	2.66E-04
10063.57656	4.75E-04	0.08782	10062.43281	3.39E-04
9057.9125	4.98E-04	0.08832	9061.05703	2.13E-04
8052.31172	6.11E-04	0.08893	8051.875	4.12E-04
7246.45938	4.30E-04	0.08936	7248.22891	3.63E-04
6610.61953	1.02E-04	0.08946	6616.32188	4.45E-04
6207.88203	1.15E-04	0.08958	6212.69453	2.70E-04
5418.05156	7.36E-05	0.08965	5421.89688	2.04E-04
5414.33984	1.75E-05	0.08967	5418.21445	4.34E-05
4814.6082	2.68E-04	0.08993	4817.72734	3.46E-04
3836.47852	5.95E-04	0.09053	3838.37148	5.44E-04

3494.04219	1.90E-04	0.09072	3495.66953	3.05E-04
2894.45156	6.10E-04	0.09133	2895.50859	5.37E-04
2502.34395	6.01E-04	0.09193	2503.08535	5.13E-04
2074.875	5.12E-04	0.09244	2075.37363	5.18E-04
1612.56572	9.91E-04	0.09343	1612.85254	9.92E-04
1308.08252	7.20E-04	0.09415	1308.2748	8.43E-04
1047.12588	8.86E-04	0.09504	1047.26055	0.0012
828.28066	9.26E-04	0.09597	828.37891	0.00144
674.32632	8.82E-04	0.09685	674.40693	0.00167
553.25195	7.98E-04	0.09765	553.31636	0.00155
433.49277	0.00144	0.09908	433.53887	0.00229
349.86316	0.0012	0.10029	349.89597	0.0018
283.06643	0.001	0.10129	283.08953	0.00153
226.89377	0.00101	0.1023	226.90977	0.00157
183.23713	9.53E-04	0.10325	183.24873	0.00175
151.17155	0.00101	0.10426	151.18074	0.00227
120.97344	0.00119	0.10545	120.98165	0.00452
95.42991	0.00188	0.10734	95.43768	0.00795
77.16335	0.00293	0.11026	77.16961	0.00719
69.06884	0.00232	0.11259	69.07402	0.00334
62.52047	0.00255	0.11514	62.52469	0.0027
55.78173	0.00331	0.11845	55.78495	0.0028
50.35594	0.00308	0.12153	50.35843	0.00234
40.26848	0.00679	0.12832	40.26982	0.00441
32.40247	0.0055	0.13382	32.40317	0.00295
26.29556	0.00426	0.13808	26.29592	0.00176
23.419	0.00198	0.14006	23.41924	6.57E-04
21.1	0.0014	0.14146	21.10018	2.40E-04
19.93934	--	0.14224	19.93947	2.16E-04
18.90328	--	0.14292	18.90339	1.64E-04
17.10948	--	0.14406	17.10956	1.43E-04
13.73443	--	0.14611	13.73447	4.05E-04
11.05097	--	0.1475	11.05098	6.04E-05
9.05797	--	0.14842	9.05797	4.01E-04
7.23756	--	0.14913	7.23756	2.43E-04
6.03017	--	0.14955	6.03017	
5.48214	--	0.1497	5.48214	
--	--	0.14975		
--		0.14975		
--		0.14975		
--		0.14975		
--		0.14975		
--		0.14975		
--		0.14975		
--		0.14783		
--		0.14217		
--		0.13535		
--		0.13015		
--		0.12823		
--		0.12667		
--		0.12509		
--		0.12283		
--		0.12118		
--		0.11951		

20-50		50-200	＞200		
	0.02791	0.01107	0.01896	0.07068	0.18024901
	0.01289	0.0247	0.01781	0.06013	0.078662897
	0.0283	0.0135	0.0377	0.0952	0.164915966
	0.0123	0.0325	0.1201	0.16588	0.005907885
				0	#DIV/0!
20-50		50-200	＞200		#VALUE!
	0.01117	0.01487	0.15814	0.18418	0
	0.02791	0.01107	0.01896	0.07068	0.18024901
	0.00864	0.0175	0.07318	0.10116	0.018189008

Cumulative Volume		Pore Diamet	intrusion volum	Cumulative Volum	Pore Diameter (nm)
%		nm	mL/g	%	nm
累计体积		3	孔容	累计体积	M3-60
	1.26E-30	370641.275	0	7.18E-31	370898.375
	0.01039	302145.025	0.00117	0.00117	301727.45
	0.01836	258826.5	0.00102	0.00219	258269.775
	0.02557	226555.2	7.31E-04	0.00293	226670.55
	0.0323	200643.413	4.69E-04	0.00339	200925.5125
	0.03693	181320.96	3.59E-04	0.00375	181325.4125
	0.04761	144415.025	0.00108	0.00483	144811.175
	0.05422	120476.888	4.28E-04	0.00526	120674.675
	0.05962	100574.519	5.52E-04	0.00581	100697.92
	0.06226	90316.2875	2.35E-04	0.00604	90289.00625
	0.06715	73042.9125	4.55E-04	0.0065	73078.15625
	0.06979	60746	3.17E-04	0.00682	60716.09375
	0.07158	52020.7375	2.90E-04	0.00711	52048.63125
	0.0728	45498.2875	2.90E-04	0.0074	45453.08125
	0.07444	36339.7438	5.38E-04	0.00793	36363.05938
	0.07543	30291.4906	1.50E-04	0.00904	30276.59688
	0.07585	27944.0719	1.24E-04	0.00916	27939.82188
	0.07616	25940.6297	1.10E-04	0.00927	25938.51406
	0.07645	24215.4719	2.07E-04	0.00948	24204.48281
	0.07696	21359.4453	2.07E-04	0.00969	21366.06094
	0.07737	19110.2844	4.55E-04	0.01014	19107.94219
	0.07776	17288.6031	2.35E-04	0.01038	17289.66719
	0.07812	15448.0828	3.31E-04	0.01071	15449.60938
	0.07846	13963.3953	2.35E-04	0.01094	13965.45781
	0.07885	12488.4305	5.11E-04	0.01145	12484.72656
	0.07912	11319.8422	4.42E-04	0.0119	11320.03359
	0.07945	10062.1156	5.38E-04	0.01243	10061.975
	0.10072	9058.46094	2.48E-04	0.01268	9059.50625
	0.10113	8052.5125	3.73E-04	0.01305	8052.95234
	0.10149	7247.63281	2.76E-04	0.01333	7247.13438
	0.10194	6524.04961	6.86E-05	0.0134	6503.08
	0.10221	6420.17813	9.23E-06	0.01341	5556.98438
	0.10241	5550.38594	1.55E-04	0.01356	5060.46328
	0.10245	5208.71602	6.93E-05	0.01363	3732.91875
	0.1028	4808.18359	1.25E-04	0.01376	3469.81523
	0.10334	3842.64141	4.54E-04	0.01421	2904.81777

0.10365	3481.55234	7.27E-04	0.01494	2484.01465
0.10419	2886.41953	0.00127	0.01621	2043.07559
0.1047	2513.05234	0.00108	0.01729	1601.20938
0.10522	2058.4918	0.00131	0.01861	1326.46416
0.10621	1611.81914	0.00173	0.02034	1047.51143
0.10705	1323.76533	0.00202	0.02235	831.72139
0.10825	1055.57793	0.00284	0.02519	675.73501
0.10969	833.66152	0.0024	0.02759	552.31294
0.11136	677.84463	0.00211	0.0297	433.6165
0.11291	553.64663	0.00202	0.03172	349.46592
0.1152	433.04951	0.0021	0.03383	283.58843
0.117	350.15276	0.00145	0.03528	226.47698
0.11853	284.01211	0.00128	0.03656	183.3078
0.1201	226.88589	0.00121	0.03776	151.11057
0.12184	183.26259	0.00107	0.03883	120.99015
0.12411	151.18646	9.66E-04	0.0398	95.43224
0.12864	120.95447	0.00116	0.04096	77.14342
0.13658	95.42617	0.00153	0.04249	69.05389
0.14378	77.16846	0.00206	0.04454	62.50186
0.14712	69.06078	0.0017	0.04624	55.77131
0.14982	62.49084	0.00209	0.04833	50.35392
0.15262	55.76524	0.00296	0.05128	40.27073
0.15497	50.34894	0.00313	0.05441	32.40158
0.15938	40.26852	0.00732	0.06173	26.29302
0.16232	32.38406	0.00709	0.06882	23.41805
0.16408	26.30223	0.00572	0.07453	21.09201
0.16474	23.41998	0.00279	0.07733	19.94975
0.16498	21.09891	0.00232	0.07964	18.90033
0.16519	19.93939	0.00118	0.08082	17.10492
0.16536	18.90309	0.00105	0.08187	13.73178
0.1655	17.10345	0.00186	0.08373	11.05185
0.16591	13.73355	0.00345	0.08718	9.05923
0.16597	11.05019	0.00262	0.0898	7.23726
0.16597	9.05748	0.00184	0.09164	6.03006
0.16597	7.23752	0.00177	0.09341	5.48196
0.16597	6.03	0.00132	0.09473	
0.16597	5.48174	6.63E-04	0.09539	
0.16565		9.97E-05	0.09549	
0.16565			0.09549	
0.16565			0.09549	
0.16565			0.09549	
0.16565			0.09549	
0.16565			0.09507	
0.16565			0.09379	
0.16565			0.08894	
0.16565			0.08306	
0.16261			0.07685	
0.15459			0.07284	
0.14839			0.07146	
0.14248			0.06992	
0.13978			0.06874	
0.13737			0.06741	
0.13585			0.06651	
0.13421			0.06536	

0.394878325	0.156621392	0.268251273
0.214368867	0.410776651	0.296191585
0.297268908	0.141806723	0.396008403
0.074149988	0.195924765	0.724017362
#DIV/0!	#DIV/0!	#DIV/0!
#VALUE!	#VALUE!	#VALUE!
0.060647193	0.080736236	0.858616571
0.394878325	0.156621392	0.268251273
0.085409253	0.172993278	0.723408462

intrusion volume	Cumulative Vo	Pore Diameter	intrusion	Cumulative Volume (mL/g)
mL/g	%	nm	mL/g	%
孔容	累计体积	M3-120	孔容	累计体积
0	6.63E-31	369651.1	0	1.53E-30
5.61E-04	5.61E-04	301874.2	0.00109	0.00109
3.19E-04	8.80E-04	259154.525	6.20E-04	0.00171
2.04E-04	0.00108	226474.525	5.02E-04	0.00221
2.55E-04	0.00134	201476.3375	3.84E-04	0.0026
1.78E-04	0.00152	181242.0875	2.66E-04	0.00286
3.06E-04	0.00182	144419.5875	5.31E-04	0.00339
2.17E-04	0.00204	120544.475	4.43E-04	0.00384
1.66E-04	0.00221	100656.475	2.66E-04	0.0041
1.15E-04	0.00232	90236.725	1.48E-04	0.00425
2.17E-04	0.00254	73028.83125	2.95E-04	0.00455
2.17E-04	0.00275	60775.58125	2.36E-04	0.00478
1.02E-04	0.00286	52022.72	1.77E-04	0.00496
1.78E-04	0.00303	45511.7375	8.86E-05	0.00505
1.40E-04	0.00317	36364.64375	3.25E-04	0.00537
1.27E-04	0.0033	30277.425	2.66E-04	0.00564
1.02E-04	0.0034	27937.96563	1.48E-04	0.00579
5.10E-05	0.00346	25941.02813	1.18E-04	0.0059
5.10E-05	0.00351	24214.45781	8.86E-05	0.00599
8.92E-05	0.0036	21357.28	1.77E-04	0.00617
5.10E-05	0.00365	19112.46875	2.07E-04	0.00638
6.37E-05	0.00371	17289.67031	1.48E-04	0.00652
8.92E-05	0.0038	15449.31094	1.48E-04	0.00667
7.65E-05	0.00388	13965.31563	2.07E-04	0.00688
7.65E-05	0.00395	12485.51797	3.54E-04	0.00723
1.02E-04	0.00405	11319.82422	1.48E-04	0.00738
8.92E-05	0.00414	10065.23906	2.07E-04	0.00759
1.15E-04	0.00426	9057.58516	2.07E-04	0.00779
1.02E-04	0.00436	8054.14375	2.36E-04	0.00803
2.80E-04	0.00464	7246.53203	2.36E-04	0.00827
2.15E-05	0.00466	6595.78906	0	0.00827
0	0.00466	6517.30273	1.59E-06	0.00827
0	0.00466	5478.73984	7.18E-05	0.00834
1.70E-04	0.00483	4784.23164	8.62E-05	0.00843
6.38E-05	0.0049	3759.13477	1.74E-04	0.0086
1.61E-04	0.00506	3435.56016	8.68E-05	0.00869

2.51E-04	0.00531	2890.70859	2.23E-04	0.00891
2.40E-04	0.00555	2486.76152	2.40E-04	0.00915
5.85E-04	0.00613	2079.83828	2.35E-04	0.00938
7.75E-04	0.00691	1601.24912	3.93E-04	0.00978
0.00138	0.00828	1326.10449	3.89E-04	0.01017
0.00146	0.00974	1045.81084	5.35E-04	0.0107
0.00127	0.01101	834.95	4.95E-04	0.0112
0.00116	0.01217	678.48745	4.28E-04	0.01162
0.00191	0.01408	552.29517	4.06E-04	0.01203
0.00162	0.0157	432.7229	3.08E-04	0.01234
9.73E-04	0.01668	349.25593	5.45E-04	0.01288
0.00113	0.01781	283.74402	6.03E-04	0.01349
0.00116	0.01897	226.81414	9.39E-04	0.01442
0.00134	0.02031	183.28975	9.80E-04	0.0154
0.00228	0.02259	151.13096	0.0012	0.01661
0.00537	0.02796	120.80088	0.00285	0.01946
0.00664	0.0346	95.33477	0.00752	0.02698
0.00303	0.03763	77.06953	0.00826	0.03524
0.00242	0.04005	69.01639	0.00373	0.03896
0.00246	0.04251	62.48781	0.00284	0.04181
0.00194	0.04445	55.73676	0.00319	0.045
0.0039	0.04835	50.32144	0.00263	0.04763
0.00317	0.05152	40.25149	0.00552	0.05315
0.00232	0.05384	32.39168	0.00383	0.05698
9.65E-04	0.0548	26.28543	0.00251	0.05949
5.97E-04	0.0554	23.40205	0.00104	0.06053
3.50E-04	0.05575	21.08994	7.15E-04	0.06124
2.93E-04	0.05604	19.93639	4.39E-04	0.06168
4.97E-04	0.05654	18.89777	3.14E-04	0.062
8.07E-04	0.05735	17.10804	4.29E-04	0.06242
6.47E-04	0.05799	13.72933	0.00118	0.0636
6.57E-04	0.05865	11.04603	6.76E-04	0.06428
8.19E-04	0.05947	9.05922	3.18E-04	0.0646
5.19E-04	0.05999	7.23656	2.92E-04	0.06489
1.46E-04	0.06014	6.02947	3.62E-04	0.06525

18.02%	39.49%	15.66%	26.83%
7.87%	21.44%	41.08%	29.62%
16.49%	29.73%	14.18%	39.60%
0.59%	7.41%	19.59%	72.40%
0.00%	6.06%	8.07%	85.86%
1.82%	8.54%	17.30%	72.34%
