# Supplementary material for: Research on the pore distribution characteristics and strength degradation of cement-based materials under sulfate attack
Source: Sci Rep. 2025 Dec 7;16:1460. doi: 10.1038/s41598-025-31233-5 (PMC12796256; doi:10.1038/s41598-025-31233-5)
Supplement: Supplementary file 2 — Supplementary Material 2 [file 41598_2025_31233_MOESM2_ESM.pdf]

T2谱数据点	P<br>孔隙信号	M<br>孔隙信号	C<br>孔隙信号
时间(ms)	0	0	0
0.001	0	0	0
0.0011	0	0	0
0.0012	0	0	0
0.0013	0	0	0
0.0014	0	0	0
0.0015	0	0	0
0.0016	0	0	0
0.0018	0	0	0
0.0019	0	0	0
0.0021	0	0	0
0.0022	0	0	0
0.0024	0	0	0
0.0026	0	0	0
0.0029	0	0	0
0.0031	0	0	0
0.0034	0	0	0
0.0037	0	0	0
0.004	0	0	0
0.0043	0	0	0
0.0047	0	0	0
0.0051	0	0	0
0.0055	0	0	0
0.0059	0	0	0
0.0064	0	0	0
0.007	0.0002	0.0001	0.0001
0.0076	0.0006	0.0002	0.0002
0.0082	0.002	0.0005	0.0007
0.0089	0.0056	0.0015	0.0021
0.0097	0.0149	0.0041	0.0055
0.0105	0.0362	0.0099	0.0135
0.0114	0.0824	0.0226	0.0307
0.0123	0.1759	0.0483	0.0654
0.0134	0.3539	0.0971	0.1317
0.0145	0.6743	0.185	0.2509
0.0157	1.2221	0.3354	0.4546
0.017	2.1149	0.5804	0.7867
0.0185	3.5074	0.9625	1.3045
0.02	5.5928	1.5349	2.0796
0.0217	8.6016	2.3607	3.1973
0.0235	12.796	3.5122	4.7542
0.0255	18.4611	5.0677	6.8547
0.0277	25.8936	7.109	9.6065
0.03	35.3875	9.7172	13.1146
0.0326	47.2202	12.9691	17.4761
0.0353	61.6381	16.9329	22.7738
0.0383	78.8433	21.6648	29.071
0.0415	98.9832	27.2059	36.4071
0.045	122.1412	33.5793	44.7942
0.0488	148.331	40.7887	54.2141
0.0529	177.4932	48.8166	64.6179
0.0574	209.4937	57.6247	75.9257

T2谱数据点<br>时间(ms)	M硫酸盐侵蚀0
0.001	0
0.0011	0
0.0012	0
0.0013	0
0.0014	0
0.0015	0
0.0016	0
0.0018	0
0.0019	0
0.0021	0
0.0022	0
0.0024	0
0.0026	0
0.0029	0
0.0031	0
0.0034	0
0.0037	0
0.004	0
0.0043	0
0.0047	0
0.0051	0
0.0055	0
0.0059	0
0.0064	0
0.007	0
0.0076	0.0001
0.0082	0.0002
0.0089	0.0005
0.0097	0.0015
0.0105	0.0041
0.0114	0.0099
0.0123	0.0226
0.0134	0.0483
0.0145	0.0971
0.0157	0.185
0.017	0.3354
0.0185	0.5804
0.02	0.9625
0.0217	1.5349
0.0235	2.3607
0.0255	3.5122
0.0277	5.0677
0.03	7.109
0.0326	9.7172
0.0353	12.9691
0.0383	16.9329
0.0415	21.6648
0.045	27.2059
0.0488	33.5793
0.0529	40.7887
0.0574	48.8166
0.0622	57.6247

0.0622	244.1251	67.153	88.0275	0.0675	67.153
0.0675	281.1099	77.3218	100.7858	0.0732	77.3218
0.0732	320.1051	88.0322	114.0384	0.0793	88.0322
0.0793	360.7081	99.1686	127.6023	0.086	99.1686
0.086	402.4646	110.6007	141.2782	0.0933	110.6007
0.0933	444.8767	122.1868	154.8551	0.1012	122.1868
0.1012	487.4123	133.7762	168.1159	0.1097	133.7762
0.1097	529.5148	145.2129	180.8423	0.119	145.2129
0.119	570.6139	156.3389	192.8202	0.129	156.3389
0.129	610.136	166.9972	203.8448	0.1399	166.9972
0.1399	647.5157	177.0357	213.7256	0.1517	177.0357
0.1517	682.2065	186.3099	222.2908	0.1645	186.3099
0.1645	713.6922	194.6863	229.3914	0.1783	194.6863
0.1783	741.4971	202.0451	234.9047	0.1934	202.0451
0.1934	765.1967	208.2826	238.7369	0.2097	208.2826
0.2097	784.4264	213.3137	240.8258	0.2274	213.3137
0.2274	798.8902	217.0733	241.1416	0.2466	217.0733
0.2466	808.3673	219.5181	239.6878	0.2674	219.5181
0.2674	812.7175	220.6269	236.501	0.2899	220.6269
0.2899	811.8849	220.4015	231.65	0.3144	220.4015
0.3144	805.8993	218.8663	225.2337	0.3409	218.8663
0.3409	794.8763	216.0674	217.3791	0.3697	216.0674
0.3697	779.0146	212.0715	208.2377	0.4009	212.0715
0.4009	758.5919	206.9643	197.9822	0.4347	206.9643
0.4347	733.9585	200.8478	186.8019	0.4714	200.8478
0.4714	705.5294	193.8382	174.8985	0.5111	193.8382
0.5111	673.7744	186.0625	162.4808	0.5543	186.0625
0.5543	639.2077	177.6557	149.7602	0.601	177.6557
0.601	602.3755	168.7575	136.9453	0.6517	168.7575
0.6517	563.8435	159.5084	124.2372	0.7067	159.5084
0.7067	524.184	150.0475	111.8246	0.7663	150.0475
0.7663	483.9632	140.5085	99.88	0.831	140.5085
0.831	443.7281	131.0173	88.5558	0.9011	131.0173
0.9011	403.9953	121.6897	77.9812	0.9771	121.6897
0.9771	365.2406	112.6289	68.26	1.0596	112.6289
1.0596	327.8893	103.9244	59.4692	1.149	103.9244
1.149	292.3093	95.6504	51.6586	1.2459	95.6504
1.2459	258.8052	87.8655	44.8511	1.351	87.8655
1.351	227.6155	80.6124	39.0439	1.465	80.6124
1.465	198.9109	73.918	34.2108	1.5886	73.918
1.5886	172.7954	67.7949	30.305	1.7226	67.7949
1.7226	149.3095	62.2419	27.2623	1.8679	62.2419
1.8679	128.4345	57.2457	25.0048	2.0255	57.2457
2.0255	110.0994	52.7831	23.4456	2.1964	52.7831
2.1964	94.1886	48.8225	22.4916	2.3817	48.8225
2.3817	80.5497	45.3264	22.0486	2.5826	45.3264
2.5826	69.0031	42.2533	22.0237	2.8005	42.2533
2.8005	59.3505	39.5599	22.329	3.0368	39.5599
3.0368	51.3833	37.2027	22.8836	3.293	37.2027
3.293	44.8902	35.1397	23.616	3.5708	35.1397
3.5708	39.6642	33.3319	24.4644	3.872	33.3319
3.872	35.5078	31.7436	25.3783	4.1987	31.7436
4.1987	32.2375	30.3437	26.3175	4.5529	30.3437
4.5529	29.687	29.1058	27.2526	4.937	29.1058

4.937	27.709	28.0081	28.1632	5.3536	28.0081
5.3536	26.1758	27.0329	29.0375	5.8052	27.0329
5.8052	24.9799	26.1669	29.8704	6.295	26.1669
6.295	24.0324	25.3999	30.6624	6.8261	25.3999
6.8261	23.2624	24.7244	31.4179	7.402	24.7244
7.402	22.6145	24.135	32.1437	8.0264	24.135
8.0264	22.0472	23.6274	32.8479	8.7036	23.6274
8.7036	21.5308	23.198	33.5382	9.4379	23.198
9.4379	21.0448	22.8432	34.2218	10.2341	22.8432
10.2341	20.5764	22.559	34.9039	11.0975	22.559
11.0975	20.118	22.3403	35.5874	12.0338	22.3403
12.0338	19.6664	22.1813	36.2727	13.049	22.1813
13.049	19.2205	22.0747	36.9579	14.1499	22.0747
14.1499	18.7807	22.0121	37.6384	15.3437	22.0121
15.3437	18.348	21.984	38.3076	16.6382	21.984
16.6382	17.923	21.98	38.9577	18.0419	21.98
18.0419	17.5061	21.9891	39.5797	19.564	21.9891
19.564	17.0965	22	40.1647	21.2145	22
21.2145	16.6929	22.0015	40.704	23.0043	22.0015
23.0043	16.2931	21.983	41.1907	24.9451	21.983
24.9451	15.8943	21.9345	41.6195	27.0496	21.9345
27.0496	15.4933	21.8474	41.988	29.3317	21.8474
29.3317	15.0869	21.7148	42.2966	31.8063	21.7148
31.8063	14.672	21.5312	42.5494	34.4896	21.5312
34.4896	14.246	21.2936	42.754	37.3994	21.2936
37.3994	13.807	21.0007	42.9214	40.5546	21.0007
40.5546	13.3537	20.6536	43.0663	43.976	20.6536
43.976	12.8862	20.2555	43.2063	47.6861	20.2555
47.6861	12.4052	19.8116	43.3614	51.7092	19.8116
51.7092	11.9128	19.3288	43.5534	56.0717	19.3288
56.0717	11.4119	18.8159	43.8049	60.8022	18.8159
60.8022	10.9065	18.2824	44.1385	65.9319	18.2824
65.9319	10.401	17.7391	44.5757	71.4943	17.7391
71.4943	9.9005	17.1971	45.1356	77.526	17.1971
77.526	9.4107	16.6674	45.8341	84.0665	16.6674
84.0665	8.9368	16.1608	46.6826	91.1589	16.1608
91.1589	8.4844	15.6872	47.6872	98.8496	15.6872
98.8496	8.0582	15.255	48.8477	107.1891	15.255
107.1891	7.6627	14.8713	50.1572	116.2322	14.8713
116.2322	7.3011	14.5411	51.6012	126.0383	14.5411
126.0383	6.9759	14.2672	53.1583	136.6716	14.2672
136.6716	6.6884	14.0501	54.7997	148.2021	14.0501
148.2021	6.4387	13.8879	56.49	160.7053	13.8879
160.7053	6.2256	13.7766	58.1885	174.2633	13.7766
174.2633	6.0469	13.7099	59.8503	188.9652	13.7099
188.9652	5.8995	13.6798	61.4279	204.9075	13.6798
204.9075	5.7792	13.6771	62.873	222.1947	13.6771
222.1947	5.6813	13.6915	64.1389	240.9404	13.6915
240.9404	5.601	13.7124	65.1817	261.2675	13.7124
261.2675	5.5328	13.7294	65.9626	283.3096	13.7294
283.3096	5.4719	13.7324	66.449	307.2113	13.7324
307.2113	5.4133	13.7125	66.6156	333.1295	13.7125
333.1295	5.3528	13.662	66.4455	361.2343	13.662
361.2343	5.2867	13.5747	65.9298	391.7101	13.5747

391.7101	5.2118	13.446	65.0682	424.7572	13.446
424.7572	5.1259	13.2731	63.8679	460.5922	13.2731
460.5922	5.0274	13.0545	62.3431	499.4505	13.0545
499.4505	4.9153	12.7904	60.5143	541.5871	12.7904
541.5871	4.7894	12.4824	58.4066	587.2787	12.4824
587.2787	4.6498	12.133	56.0489	636.825	12.133
636.825	4.4974	11.7458	53.4729	690.5514	11.7458
690.5514	4.3333	11.3248	50.7116	748.8104	11.3248
748.8104	4.1587	10.8748	47.7983	811.9845	10.8748
811.9845	3.9753	10.4006	44.7663	880.4884	10.4006
880.4884	3.7846	9.9072	41.6474	954.7716	9.9072
954.7716	3.5886	9.3995	38.472	1035.3218	9.3995
1035.3218	3.3887	8.8822	35.2681	1122.6678	8.8822
1122.6678	3.1866	8.3599	32.0614	1217.3827	8.3599
1217.3827	2.984	7.8366	28.8748	1320.0884	7.8366
1320.0884	2.7822	7.3161	25.7285	1431.4589	7.3161
1431.4589	2.5824	6.8017	22.6402	1552.2254	6.8017
1552.2254	2.3859	6.2962	19.6247	1683.1804	6.2962
1683.1804	2.1935	5.8022	16.6941	1825.1835	5.8022
1825.1835	2.0061	5.3218	13.8586	1979.1669	5.3218
1979.1669	1.8244	4.8565	11.1258	2146.1412	4.8565
2146.1412	1.6489	4.4077	8.5016	2327.2025	4.4077
2327.2025	1.4801	3.9765	5.9897	2523.5392	3.9765
2523.5392	1.3182	3.5635	3.5928	2736.44	3.5635
2736.44	1.1635	3.1691	1.3118	2967.3024	3.1691
2967.3024	1.016	2.7936	0	3217.6417	2.7936
3217.6417	0.8758	2.437	0	3489.1012	2.437
3489.1012	0.7428	2.0991	0	3783.4626	2.0991
3783.4626	0.617	1.7796	0	4102.6581	1.7796
4102.6581	0.4982	1.4782	0	4448.7828	1.4782
4448.7828	0.3863	1.1942	0	4824.1087	1.1942
4824.1087	0.2809	0.9273	0	5231.0993	0.9273
5231.0993	0.182	0.6767	0	5672.4261	0.6767
5672.4261	0.0891	0.4417	0	6150.9858	0.4417
6150.9858	0.0022	0.2217	0	6669.9197	0.2217
6669.9197	0	0.016	0	7232.6339	0.016
7232.6339	0	0	0	7842.8221	0
7842.8221	0	0	0	8504.4893	0
8504.4893	0	0	0	9221.9788	0
9221.9788	0	0	0	10000	0
10000					

M硫酸盐侵蚀30	M硫酸盐侵蚀90	M硫酸盐侵蚀150
0.0068	0.0064	0.0065
0.0152	0.0142	0.0144
0.0322	0.0301	0.0306
0.0648	0.0606	0.0616
0.1245	0.1166	0.1184
0.2291	0.2144	0.2178
0.4046	0.3787	0.3847
0.6878	0.6438	0.6539
1.1283	1.0562	1.0726
1.7904	1.6761	1.702
2.754	2.5786	2.6181
4.1153	3.8538	3.9122
5.9852	5.6063	5.6896
8.4872	7.9525	8.0679
11.7537	11.0179	11.1729
15.9213	14.9328	15.1342
21.1249	19.827	20.08
27.4915	25.8242	26.1308
35.134	33.0367	33.3934
44.1443	41.559	41.9551
54.5888	51.4636	51.8782
66.503	62.796	63.1958
79.8884	75.5719	75.9085
94.7109	89.7742	89.9827
110.8996	105.3525	105.3498
128.3479	122.2229	121.907
146.915	140.2687	139.519
166.4297	159.3422	158.0211
186.6931	179.2682	177.223
207.4843	199.8464	196.9126
228.5641	220.8562	216.8611
249.681	242.0606	236.8282
270.5759	263.2108	256.5663
290.9875	284.0514	275.8263
310.657	304.3246	294.3619
329.3329	323.7755	311.9339
346.7755	342.1562	328.3148
362.7605	359.2306	343.2922
377.0828	374.7779	356.6726
389.56	388.5966	368.2838
400.0345	400.5074	377.9778
408.376	410.3559	385.6328
414.4838	418.0152	391.1552
418.2873	423.3871	394.4802
419.7476	426.404	395.5732
418.8576	427.029	394.43
415.642	425.257	391.0766
410.1567	421.1141	385.5688
402.4878	414.6573	377.9914
392.7503	405.9736	368.4564
381.0859	395.1786	357.1015
367.6609	382.415	344.088

P
0~0.02
0.02~0.05
0.05~0.2
＞0.2
合计

M-0
0~0.02
0.02~0.05
0.05~0.2
＞0.2
合计

P	85.87%
M	71.14%
C	61.50%

0	71.14%
30	77.94%
90	83.36%
150	78.59%

352.6636	367.8502	329.5975
336.3005	351.674	313.8296
318.7939	334.0955	296.9982
300.3771	315.3399	279.3281
281.2914	295.6449	261.0506
261.7811	275.2569	242.4002
242.0898	254.4269	223.6099
222.4558	233.4057	204.907
203.1079	212.4402	186.5094
184.2611	191.7684	168.6209
166.1125	171.6155	151.4279
148.8375	152.1894	135.0956
132.587	133.6773	119.765
117.484	116.2417	105.5503
103.6217	100.0185	92.5371
91.0625	85.1139	80.7814
79.8375	71.6037	70.3091
69.9469	59.533	61.1172
61.3623	48.9162	53.1752
54.0293	39.7387	46.4283
47.8711	31.9595	40.8008
42.7933	25.5141	36.2002
38.6891	20.3183	32.5229
35.4443	16.2729	29.6582
32.943	13.2675	27.4942
31.0724	11.1857	25.9222
29.7271	9.9091	24.8404
28.8124	9.3211	24.1575
28.247	9.3104	23.7949
27.9637	9.7733	23.6877
27.9101	10.6159	23.785
28.0471	11.7548	24.049
28.3479	13.1174	24.4537
28.7951	14.6424	24.9825
29.3783	16.2778	25.626
30.0912	17.9808	26.379
30.9292	19.7163	27.2386
31.8868	21.455	28.2014
32.9559	23.1731	29.262
34.1247	24.8501	30.4119
35.3773	26.4691	31.6387
36.693	28.0151	32.926
38.048	29.4754	34.2538
39.4152	30.8394	35.5992
40.7665	32.0981	36.9378
42.0737	33.2449	38.2446
43.3098	34.2751	39.4955
44.4511	35.1866	40.6684
45.4773	35.9795	41.7441
46.373	36.6563	42.7075
47.1279	37.2217	43.5472
47.7369	37.682	44.2567
48.2002	38.0449	44.8334
48.5221	38.3192	45.2788

48.7112	38.5138	45.5977
48.7788	38.6375	45.7975
48.7381	38.6983	45.8875
48.6038	38.7033	45.878
48.3902	38.658	45.7798
48.1115	38.5665	45.6033
47.7803	38.4313	45.3583
47.4075	38.2533	45.0534
47.0021	38.0324	44.6958
46.5707	37.7672	44.2914
46.118	37.4557	43.8447
45.6464	37.0957	43.3587
45.1566	36.6849	42.8354
44.6479	36.2212	42.2761
44.1184	35.7035	41.6811
43.5654	35.1309	41.0507
42.9859	34.504	40.3848
42.3767	33.824	39.6835
41.7349	33.0931	38.947
41.0582	32.3146	38.176
40.3445	31.4925	37.3714
39.593	30.6314	36.5347
38.8033	29.7366	35.6681
37.9762	28.8136	34.7738
37.113	27.8683	33.8549
36.2159	26.9066	32.9147
35.2879	25.9343	31.9565
34.3324	24.9569	30.9844
33.3533	23.9799	30.002
32.3548	23.0082	29.0135
31.3415	22.0464	28.0227
30.3178	21.0986	27.0336
29.2884	20.1684	26.0497
28.2578	19.259	25.0747
27.2302	18.3731	24.1118
26.2097	17.5128	23.1641
25.2002	16.6801	22.2341
24.2051	15.8761	21.3244
23.2276	15.1021	20.437
22.2705	14.3586	19.5737
21.3362	13.646	18.7361
20.4268	12.9644	17.9254
19.5441	12.3138	17.1424
18.6894	11.6937	16.3879
17.8639	11.1038	15.6624
17.0683	10.5433	14.9659
16.3031	10.0115	14.2987
15.5687	9.5076	13.6605
14.8652	9.0307	13.0511
14.1924	8.5798	12.47
13.5499	8.1539	11.9167
12.9375	7.752	11.3905
12.3544	7.3731	10.8908
11.8001	7.0161	10.4168

11.2738	6.68	9.9676
10.7746	6.3638	9.5424
10.3017	6.0665	9.1403
9.8541	5.7872	8.7603
9.4309	5.5248	8.4016
9.0311	5.2784	8.0631
8.6537	5.0473	7.7441
8.2978	4.8305	7.4436
7.9623	4.6272	7.1607
7.6464	4.4367	6.8945
7.349	4.2582	6.6442
7.0693	4.0909	6.4089
6.8063	3.9344	6.188
6.5591	3.7878	5.9805
6.327	3.6506	5.7857
6.1091	3.5222	5.603
5.9046	3.4021	5.4317
5.7127	3.2898	5.271
5.5329	3.1847	5.1205
5.3643	3.0865	4.9794
5.2062	2.9947	4.8473
5.0582	2.9088	4.7236
4.9196	2.8286	4.6078
4.7898	2.7536	4.4994
4.6683	2.6836	4.398
4.5546	2.6181	4.3031
4.4482	2.557	4.2143
4.3486	2.4999	4.1313
4.2555	2.4465	4.0537
4.1685	2.3967	3.9811
4.0871	2.3501	3.9133
4.011	2.3067	3.8499
3.9398	2.2661	3.7906
3.8734	2.2282	3.7352
3.8112	2.1929	3.6835
3.7532	2.1598	3.6352
3.6989	2.129	3.59
3.6483	2.1002	3.5478
3.6009	2.0734	3.5084
3.5567	2.0483	3.4717

		M			C		
21.4073	0.858692905	0~0.02	6.3169	0.71140267	0~0.02	6.931	0.615022849
2.2848	0.091648249	0.02~0.05	0.8881	0.10001689	0.02~0.05	0.4649	0.041252939
0.6236	0.025013939	0.05~0.2	0.5539	0.06237964	0.05~0.2	0.5064	0.044935445
0.6144	0.024644907	＞0.2	1.1206	0.1262008	＞0.2	3.3672	0.298788766
24.9301		合计	8.8795		合计	11	

		M-30			M-90		
6.3169	0.711402669	0~0.02	5.9236	0.77939029	0~0.02	5.7601	0.833552812
0.8881	0.100016893	0.02~0.05	0.2978	0.03918266	0.02~0.05	0.1976	0.028594996
0.5539	0.062379638	0.05~0.2	0.3141	0.04132732	0.05~0.2	0.1973	0.028551582
1.1206	0.1262008	＞0.2	1.0648	0.14009973	＞0.2	0.7553	0.109300609
8.8795		合计	7.6003		合计	6.9103	

9.17%	2.50%	2.46%
10.00%	6.24%	12.62%
4.13%	4.49%	29.88%

10.00%	6.24%	12.62%
3.92%	4.13%	14.01%
2.86%	2.86%	10.93%
3.62%	3.94%	13.86%

M-150

0~0.02	11.3325	0.785882206
0.02~0.05	0.5219	0.036192537
0.05~0.2	0.5675	0.039354789
＞0.2	1.9982	0.138570468
合计	14.4201	
