# Supplementary material for: Research on the pore distribution characteristics and strength degradation of cement-based materials under sulfate attack
Source: Sci Rep. 2025 Dec 7;16:1460. doi: 10.1038/s41598-025-31233-5 (PMC12796256; doi:10.1038/s41598-025-31233-5)
Supplement: Supplementary file 4 — Supplementary Material 4 [file 41598_2025_31233_MOESM4_ESM.pdf]

	P	M	C	T2
T2	Pore signal	Pore signal	Pore signal	time(ms)
time(ms)	0	0	0	0.001
0.001	0	0	0	0.0011
0.0011	0	0	0	0.0012
0.0012	0	0	0	0.0013
0.0013	0	0	0	0.0014
0.0014	0	0	0	0.0015
0.0015	0	0	0	0.0016
0.0016	0	0	0	0.0018
0.0018	0	0	0	0.0019
0.0019	0	0	0	0.0021
0.0021	0	0	0	0.0022
0.0022	0	0	0	0.0024
0.0024	0	0	0	0.0026
0.0026	0	0	0	0.0029
0.0029	0	0	0	0.0031
0.0031	0	0	0	0.0034
0.0034	0	0	0	0.0037
0.0037	0	0	0	0.004
0.004	0	0	0	0.0043
0.0043	0	0	0	0.0047
0.0047	0	0	0	0.0051
0.0051	0	0	0	0.0055
0.0055	0	0	0	0.0059
0.0059	0	0	0	0.0064
0.0064	0	0	0	0.007
0.007	0.0002	0.0001	0.0001	0.0076
0.0076	0.0006	0.0002	0.0002	0.0082
0.0082	0.002	0.0005	0.0007	0.0089
0.0089	0.0056	0.0015	0.0021	0.0097
0.0097	0.0149	0.0041	0.0055	0.0105
0.0105	0.0362	0.0099	0.0135	0.0114
0.0114	0.0824	0.0226	0.0307	0.0123
0.0123	0.1759	0.0483	0.0654	0.0134
0.0134	0.3539	0.0971	0.1317	0.0145
0.0145	0.6743	0.185	0.2509	0.0157
0.0157	1.2221	0.3354	0.4546	0.017
0.017	2.1149	0.5804	0.7867	0.0185
0.0185	3.5074	0.9625	1.3045	0.02
0.02	5.5928	1.5349	2.0796	0.0217
0.0217	8.6016	2.3607	3.1973	0.0235
0.0235	12.796	3.5122	4.7542	0.0255
0.0255	18.4611	5.0677	6.8547	0.0277
0.0277	25.8936	7.109	9.6065	0.03
0.03	35.3875	9.7172	13.1146	0.0326
0.0326	47.2202	12.9691	17.4761	0.0353
0.0353	61.6381	16.9329	22.7738	0.0383
0.0383	78.8433	21.6648	29.071	0.0415
0.0415	98.9832	27.2059	36.4071	0.045
0.045	122.1412	33.5793	44.7942	0.0488
0.0488	148.331	40.7887	54.2141	0.0529
0.0529	177.4932	48.8166	64.6179	0.0574
0.0574	209.4937	57.6247	75.9257	0.0622

0.0622	244.1251	67.153	88.0275	0.0675
0.0675	281.1099	77.3218	100.7858	0.0732
0.0732	320.1051	88.0322	114.0384	0.0793
0.0793	360.7081	99.1686	127.6023	0.086
0.086	402.4646	110.6007	141.2782	0.0933
0.0933	444.8767	122.1868	154.8551	0.1012
0.1012	487.4123	133.7762	168.1159	0.1097
0.1097	529.5148	145.2129	180.8423	0.119
0.119	570.6139	156.3389	192.8202	0.129
0.129	610.136	166.9972	203.8448	0.1399
0.1399	647.5157	177.0357	213.7256	0.1517
0.1517	682.2065	186.3099	222.2908	0.1645
0.1645	713.6922	194.6863	229.3914	0.1783
0.1783	741.4971	202.0451	234.9047	0.1934
0.1934	765.1967	208.2826	238.7369	0.2097
0.2097	784.4264	213.3137	240.8258	0.2274
0.2274	798.8902	217.0733	241.1416	0.2466
0.2466	808.3673	219.5181	239.6878	0.2674
0.2674	812.7175	220.6269	236.501	0.2899
0.2899	811.8849	220.4015	231.65	0.3144
0.3144	805.8993	218.8663	225.2337	0.3409
0.3409	794.8763	216.0674	217.3791	0.3697
0.3697	779.0146	212.0715	208.2377	0.4009
0.4009	758.5919	206.9643	197.9822	0.4347
0.4347	733.9585	200.8478	186.8019	0.4714
0.4714	705.5294	193.8382	174.8985	0.5111
0.5111	673.7744	186.0625	162.4808	0.5543
0.5543	639.2077	177.6557	149.7602	0.601
0.601	602.3755	168.7575	136.9453	0.6517
0.6517	563.8435	159.5084	124.2372	0.7067
0.7067	524.184	150.0475	111.8246	0.7663
0.7663	483.9632	140.5085	99.88	0.831
0.831	443.7281	131.0173	88.5558	0.9011
0.9011	403.9953	121.6897	77.9812	0.9771
0.9771	365.2406	112.6289	68.26	1.0596
1.0596	327.8893	103.9244	59.4692	1.149
1.149	292.3093	95.6504	51.6586	1.2459
1.2459	258.8052	87.8655	44.8511	1.351
1.351	227.6155	80.6124	39.0439	1.465
1.465	198.9109	73.918	34.2108	1.5886
1.5886	172.7954	67.7949	30.305	1.7226
1.7226	149.3095	62.2419	27.2623	1.8679
1.8679	128.4345	57.2457	25.0048	2.0255
2.0255	110.0994	52.7831	23.4456	2.1964
2.1964	94.1886	48.8225	22.4916	2.3817
2.3817	80.5497	45.3264	22.0486	2.5826
2.5826	69.0031	42.2533	22.0237	2.8005
2.8005	59.3505	39.5599	22.329	3.0368
3.0368	51.3833	37.2027	22.8836	3.293
3.293	44.8902	35.1397	23.616	3.5708
3.5708	39.6642	33.3319	24.4644	3.872
3.872	35.5078	31.7436	25.3783	4.1987
4.1987	32.2375	30.3437	26.3175	4.5529
4.5529	29.687	29.1058	27.2526	4.937

4.937	27.709	28.0081	28.1632	5.3536
5.3536	26.1758	27.0329	29.0375	5.8052
5.8052	24.9799	26.1669	29.8704	6.295
6.295	24.0324	25.3999	30.6624	6.8261
6.8261	23.2624	24.7244	31.4179	7.402
7.402	22.6145	24.135	32.1437	8.0264
8.0264	22.0472	23.6274	32.8479	8.7036
8.7036	21.5308	23.198	33.5382	9.4379
9.4379	21.0448	22.8432	34.2218	10.2341
10.2341	20.5764	22.559	34.9039	11.0975
11.0975	20.118	22.3403	35.5874	12.0338
12.0338	19.6664	22.1813	36.2727	13.049
13.049	19.2205	22.0747	36.9579	14.1499
14.1499	18.7807	22.0121	37.6384	15.3437
15.3437	18.348	21.984	38.3076	16.6382
16.6382	17.923	21.98	38.9577	18.0419
18.0419	17.5061	21.9891	39.5797	19.564
19.564	17.0965	22	40.1647	21.2145
21.2145	16.6929	22.0015	40.704	23.0043
23.0043	16.2931	21.983	41.1907	24.9451
24.9451	15.8943	21.9345	41.6195	27.0496
27.0496	15.4933	21.8474	41.988	29.3317
29.3317	15.0869	21.7148	42.2966	31.8063
31.8063	14.672	21.5312	42.5494	34.4896
34.4896	14.246	21.2936	42.754	37.3994
37.3994	13.807	21.0007	42.9214	40.5546
40.5546	13.3537	20.6536	43.0663	43.976
43.976	12.8862	20.2555	43.2063	47.6861
47.6861	12.4052	19.8116	43.3614	51.7092
51.7092	11.9128	19.3288	43.5534	56.0717
56.0717	11.4119	18.8159	43.8049	60.8022
60.8022	10.9065	18.2824	44.1385	65.9319
65.9319	10.401	17.7391	44.5757	71.4943
71.4943	9.9005	17.1971	45.1356	77.526
77.526	9.4107	16.6674	45.8341	84.0665
84.0665	8.9368	16.1608	46.6826	91.1589
91.1589	8.4844	15.6872	47.6872	98.8496
98.8496	8.0582	15.255	48.8477	107.1891
107.1891	7.6627	14.8713	50.1572	116.2322
116.2322	7.3011	14.5411	51.6012	126.0383
126.0383	6.9759	14.2672	53.1583	136.6716
136.6716	6.6884	14.0501	54.7997	148.2021
148.2021	6.4387	13.8879	56.49	160.7053
160.7053	6.2256	13.7766	58.1885	174.2633
174.2633	6.0469	13.7099	59.8503	188.9652
188.9652	5.8995	13.6798	61.4279	204.9075
204.9075	5.7792	13.6771	62.873	222.1947
222.1947	5.6813	13.6915	64.1389	240.9404
240.9404	5.601	13.7124	65.1817	261.2675
261.2675	5.5328	13.7294	65.9626	283.3096
283.3096	5.4719	13.7324	66.449	307.2113
307.2113	5.4133	13.7125	66.6156	333.1295
333.1295	5.3528	13.662	66.4455	361.2343
361.2343	5.2867	13.5747	65.9298	391.7101

391.7101	5.2118	13.446	65.0682	424.7572
424.7572	5.1259	13.2731	63.8679	460.5922
460.5922	5.0274	13.0545	62.3431	499.4505
499.4505	4.9153	12.7904	60.5143	541.5871
541.5871	4.7894	12.4824	58.4066	587.2787
587.2787	4.6498	12.133	56.0489	636.825
636.825	4.4974	11.7458	53.4729	690.5514
690.5514	4.3333	11.3248	50.7116	748.8104
748.8104	4.1587	10.8748	47.7983	811.9845
811.9845	3.9753	10.4006	44.7663	880.4884
880.4884	3.7846	9.9072	41.6474	954.7716
954.7716	3.5886	9.3995	38.472	1035.3218
1035.3218	3.3887	8.8822	35.2681	1122.6678
1122.6678	3.1866	8.3599	32.0614	1217.3827
1217.3827	2.984	7.8366	28.8748	1320.0884
1320.0884	2.7822	7.3161	25.7285	1431.4589
1431.4589	2.5824	6.8017	22.6402	1552.2254
1552.2254	2.3859	6.2962	19.6247	1683.1804
1683.1804	2.1935	5.8022	16.6941	1825.1835
1825.1835	2.0061	5.3218	13.8586	1979.1669
1979.1669	1.8244	4.8565	11.1258	2146.1412
2146.1412	1.6489	4.4077	8.5016	2327.2025
2327.2025	1.4801	3.9765	5.9897	2523.5392
2523.5392	1.3182	3.5635	3.5928	2736.44
2736.44	1.1635	3.1691	1.3118	2967.3024
2967.3024	1.016	2.7936	0	3217.6417
3217.6417	0.8758	2.437	0	3489.1012
3489.1012	0.7428	2.0991	0	3783.4626
3783.4626	0.617	1.7796	0	4102.6581
4102.6581	0.4982	1.4782	0	4448.7828
4448.7828	0.3863	1.1942	0	4824.1087
4824.1087	0.2809	0.9273	0	5231.0993
5231.0993	0.182	0.6767	0	5672.4261
5672.4261	0.0891	0.4417	0	6150.9858
6150.9858	0.0022	0.2217	0	6669.9197
6669.9197	0	0.016	0	7232.6339
7232.6339	0	0	0	7842.8221
7842.8221	0	0	0	8504.4893
8504.4893	0	0	0	9221.9788
9221.9788	0	0	0	10000
10000				

M Sulfate erosion 0	M Sulfate erosion 30	M Sulfate erosion 90
0	0.0068	0.0064
0	0.0152	0.0142
0	0.0322	0.0301
0	0.0648	0.0606
0	0.1245	0.1166
0	0.2291	0.2144
0	0.4046	0.3787
0	0.6878	0.6438
0	1.1283	1.0562
0	1.7904	1.6761
0	2.754	2.5786
0	4.1153	3.8538
0	5.9852	5.6063
0	8.4872	7.9525
0	11.7537	11.0179
0	15.9213	14.9328
0	21.1249	19.827
0	27.4915	25.8242
0	35.134	33.0367
0	44.1443	41.559
0	54.5888	51.4636
0	66.503	62.796
0	79.8884	75.5719
0	94.7109	89.7742
0	110.8996	105.3525
0.0001	128.3479	122.2229
0.0002	146.915	140.2687
0.0005	166.4297	159.3422
0.0015	186.6931	179.2682
0.0041	207.4843	199.8464
0.0099	228.5641	220.8562
0.0226	249.681	242.0606
0.0483	270.5759	263.2108
0.0971	290.9875	284.0514
0.185	310.657	304.3246
0.3354	329.3329	323.7755
0.5804	346.7755	342.1562
0.9625	362.7605	359.2306
1.5349	377.0828	374.7779
2.3607	389.56	388.5966
3.5122	400.0345	400.5074
5.0677	408.376	410.3559
7.109	414.4838	418.0152
9.7172	418.2873	423.3871
12.9691	419.7476	426.404
16.9329	418.8576	427.029
21.6648	415.642	425.257
27.2059	410.1567	421.1141
33.5793	402.4878	414.6573
40.7887	392.7503	405.9736
48.8166	381.0859	395.1786
57.6247	367.6609	382.415

67.153	352.6636	367.8502
77.3218	336.3005	351.674
88.0322	318.7939	334.0955
99.1686	300.3771	315.3399
110.6007	281.2914	295.6449
122.1868	261.7811	275.2569
133.7762	242.0898	254.4269
145.2129	222.4558	233.4057
156.3389	203.1079	212.4402
166.9972	184.2611	191.7684
177.0357	166.1125	171.6155
186.3099	148.8375	152.1894
194.6863	132.587	133.6773
202.0451	117.484	116.2417
208.2826	103.6217	100.0185
213.3137	91.0625	85.1139
217.0733	79.8375	71.6037
219.5181	69.9469	59.533
220.6269	61.3623	48.9162
220.4015	54.0293	39.7387
218.8663	47.8711	31.9595
216.0674	42.7933	25.5141
212.0715	38.6891	20.3183
206.9643	35.4443	16.2729
200.8478	32.943	13.2675
193.8382	31.0724	11.1857
186.0625	29.7271	9.9091
177.6557	28.8124	9.3211
168.7575	28.247	9.3104
159.5084	27.9637	9.7733
150.0475	27.9101	10.6159
140.5085	28.0471	11.7548
131.0173	28.3479	13.1174
121.6897	28.7951	14.6424
112.6289	29.3783	16.2778
103.9244	30.0912	17.9808
95.6504	30.9292	19.7163
87.8655	31.8868	21.455
80.6124	32.9559	23.1731
73.918	34.1247	24.8501
67.7949	35.3773	26.4691
62.2419	36.693	28.0151
57.2457	38.048	29.4754
52.7831	39.4152	30.8394
48.8225	40.7665	32.0981
45.3264	42.0737	33.2449
42.2533	43.3098	34.2751
39.5599	44.4511	35.1866
37.2027	45.4773	35.9795
35.1397	46.373	36.6563
33.3319	47.1279	37.2217
31.7436	47.7369	37.682
30.3437	48.2002	38.0449
29.1058	48.5221	38.3192

28.0081	48.7112	38.5138
27.0329	48.7788	38.6375
26.1669	48.7381	38.6983
25.3999	48.6038	38.7033
24.7244	48.3902	38.658
24.135	48.1115	38.5665
23.6274	47.7803	38.4313
23.198	47.4075	38.2533
22.8432	47.0021	38.0324
22.559	46.5707	37.7672
22.3403	46.118	37.4557
22.1813	45.6464	37.0957
22.0747	45.1566	36.6849
22.0121	44.6479	36.2212
21.984	44.1184	35.7035
21.98	43.5654	35.1309
21.9891	42.9859	34.504
22	42.3767	33.824
22.0015	41.7349	33.0931
21.983	41.0582	32.3146
21.9345	40.3445	31.4925
21.8474	39.593	30.6314
21.7148	38.8033	29.7366
21.5312	37.9762	28.8136
21.2936	37.113	27.8683
21.0007	36.2159	26.9066
20.6536	35.2879	25.9343
20.2555	34.3324	24.9569
19.8116	33.3533	23.9799
19.3288	32.3548	23.0082
18.8159	31.3415	22.0464
18.2824	30.3178	21.0986
17.7391	29.2884	20.1684
17.1971	28.2578	19.259
16.6674	27.2302	18.3731
16.1608	26.2097	17.5128
15.6872	25.2002	16.6801
15.255	24.2051	15.8761
14.8713	23.2276	15.1021
14.5411	22.2705	14.3586
14.2672	21.3362	13.646
14.0501	20.4268	12.9644
13.8879	19.5441	12.3138
13.7766	18.6894	11.6937
13.7099	17.8639	11.1038
13.6798	17.0683	10.5433
13.6771	16.3031	10.0115
13.6915	15.5687	9.5076
13.7124	14.8652	9.0307
13.7294	14.1924	8.5798
13.7324	13.5499	8.1539
13.7125	12.9375	7.752
13.662	12.3544	7.3731
13.5747	11.8001	7.0161

13.446	11.2738	6.68
13.2731	10.7746	6.3638
13.0545	10.3017	6.0665
12.7904	9.8541	5.7872
12.4824	9.4309	5.5248
12.133	9.0311	5.2784
11.7458	8.6537	5.0473
11.3248	8.2978	4.8305
10.8748	7.9623	4.6272
10.4006	7.6464	4.4367
9.9072	7.349	4.2582
9.3995	7.0693	4.0909
8.8822	6.8063	3.9344
8.3599	6.5591	3.7878
7.8366	6.327	3.6506
7.3161	6.1091	3.5222
6.8017	5.9046	3.4021
6.2962	5.7127	3.2898
5.8022	5.5329	3.1847
5.3218	5.3643	3.0865
4.8565	5.2062	2.9947
4.4077	5.0582	2.9088
3.9765	4.9196	2.8286
3.5635	4.7898	2.7536
3.1691	4.6683	2.6836
2.7936	4.5546	2.6181
2.437	4.4482	2.557
2.0991	4.3486	2.4999
1.7796	4.2555	2.4465
1.4782	4.1685	2.3967
1.1942	4.0871	2.3501
0.9273	4.011	2.3067
0.6767	3.9398	2.2661
0.4417	3.8734	2.2282
0.2217	3.8112	2.1929
0.016	3.7532	2.1598
0	3.6989	2.129
0	3.6483	2.1002
0	3.6009	2.0734
0	3.5567	2.0483

M Sulfate erosion 150

0.0065		P			
0.0144		0~0.02	21.4073	0.858692905	
0.0306		0.02~0.05	2.2848	0.091648249	
0.0616		0.05~0.2	0.6236	0.025013939	
0.1184		＞0.2	0.6144	0.024644907	
0.2178		sum	24.9301		
0.3847					
0.6539					
1.0726					
1.702		M-0			
2.6181		0~0.02	6.3169	0.711402669	
3.9122		0.02~0.05	0.8881	0.100016893	
5.6896		0.05~0.2	0.5539	0.062379638	
8.0679		＞0.2	1.1206	0.1262008	
11.1729		sum	8.8795		
15.1342					
20.08					
26.1308					
33.3934	P		85.87%	9.17%	2.50%
41.9551	M		71.14%	10.00%	6.24%
51.8782	C		61.50%	4.13%	4.49%
63.1958					
75.9085					
89.9827		0	71.14%	10.00%	6.24%
105.3498		30	77.94%	3.92%	4.13%
121.907		90	83.36%	2.86%	2.86%
139.519		150	78.59%	3.62%	3.94%
158.0211					
177.223					
196.9126					
216.8611					
236.8282					
256.5663					
275.8263					
294.3619					
311.9339					
328.3148					
343.2922					
356.6726					
368.2838					
377.9778					
385.6328					
391.1552					
394.4802					
395.5732					
394.43					
391.0766					
385.5688					
377.9914					
368.4564					
357.1015					
344.088					

329.5975
313.8296
296.9982
279.3281
261.0506
242.4002
223.6099
204.907
186.5094
168.6209
151.4279
135.0956
119.765
105.5503
92.5371
80.7814
70.3091
61.1172
53.1752
46.4283
40.8008
36.2002
32.5229
29.6582
27.4942
25.9222
24.8404
24.1575
23.7949
23.6877
23.785
24.049
24.4537
24.9825
25.626
26.379
27.2386
28.2014
29.262
30.4119
31.6387
32.926
34.2538
35.5992
36.9378
38.2446
39.4955
40.6684
41.7441
42.7075
43.5472
44.2567
44.8334
45.2788

45.5977
45.7975
45.8875
45.878
45.7798
45.6033
45.3583
45.0534
44.6958
44.2914
43.8447
43.3587
42.8354
42.2761
41.6811
41.0507
40.3848
39.6835
38.947
38.176
37.3714
36.5347
35.6681
34.7738
33.8549
32.9147
31.9565
30.9844
30.002
29.0135
28.0227
27.0336
26.0497
25.0747
24.1118
23.1641
22.2341
21.3244
20.437
19.5737
18.7361
17.9254
17.1424
16.3879
15.6624
14.9659
14.2987
13.6605
13.0511
12.47
11.9167
11.3905
10.8908
10.4168

9.9676
9.5424
9.1403
8.7603
8.4016
8.0631
7.7441
7.4436
7.1607
6.8945
6.6442
6.4089
6.188
5.9805
5.7857
5.603
5.4317
5.271
5.1205
4.9794
4.8473
4.7236
4.6078
4.4994
4.398
4.3031
4.2143
4.1313
4.0537
3.9811
3.9133
3.8499
3.7906
3.7352
3.6835
3.6352
3.59
3.5478
3.5084
3.4717

M			C		
0~0.02	6.3169	0.71140267	0~0.02	6.931	0.615022849
0.02~0.05	0.8881	0.10001689	0.02~0.05	0.4649	0.041252939
0.05~0.2	0.5539	0.06237964	0.05~0.2	0.5064	0.044935445
＞0.2	1.1206	0.1262008	＞0.2	3.3672	0.298788766
sum	8.8795		sum	11	

M-30			M-90			M-150	
0~0.02	5.9236	0.77939029	0~0.02	5.7601	0.833552812	0~0.02	11.3325
0.02~0.05	0.2978	0.03918266	0.02~0.05	0.1976	0.028594996	0.02~0.05	0.5219
0.05~0.2	0.3141	0.04132732	0.05~0.2	0.1973	0.028551582	0.05~0.2	0.5675
＞0.2	1.0648	0.14009973	＞0.2	0.7553	0.109300609	＞0.2	1.9982
sum	7.6003		sum	6.9103		sum	14.4201

2.46%
12.62%
29.88%

12.62%
14.01%
10.93%
13.86%

0.785882206
0.036192537
0.039354789
0.138570468
